# First-principles calculations of hybrid inorganic–organic interfaces: from state-of-the-art to best practice

**DOI:** 10.1039/d0cp06605b

**Published:** 2021-03-05

**Authors:** Oliver T. Hofmann, Egbert Zojer, Lukas Hörmann, Andreas Jeindl, Reinhard J. Maurer

**Affiliations:** Institute of Solid State Physics, Graz University of Technology, NAWI Graz Petersgasse 16/II 8010 Graz Austria o.hofmann@tugraz.at; Department of Chemistry, University of Warwick Coventry CV4 7AL UK

## Abstract

The computational characterization of inorganic–organic hybrid interfaces is arguably one of the technically most challenging applications of density functional theory. Due to the fundamentally different electronic properties of the inorganic and the organic components of a hybrid interface, the proper choice of the electronic structure method, of the algorithms to solve these methods, and of the parameters that enter these algorithms is highly non-trivial. In fact, computational choices that work well for one of the components often perform poorly for the other. As a consequence, default settings for one materials class are typically inadequate for the hybrid system, which makes calculations employing such settings inefficient and sometimes even prone to erroneous results. To address this issue, we discuss how to choose appropriate atomistic representations for the system under investigation, we highlight the role of the exchange–correlation functional and the van der Waals correction employed in the calculation and we provide tips and tricks how to efficiently converge the self-consistent field cycle and to obtain accurate geometries. We particularly focus on potentially unexpected pitfalls and the errors they incur. As a summary, we provide a list of best practice rules for interface simulations that should especially serve as a useful starting point for less experienced users and newcomers to the field.

## Abstract 2

This work highlights the challenges and problems when modelling inorganic–organic interfaces and provides practical tips and suggestions for efficient calculations.

## Graphical abstract

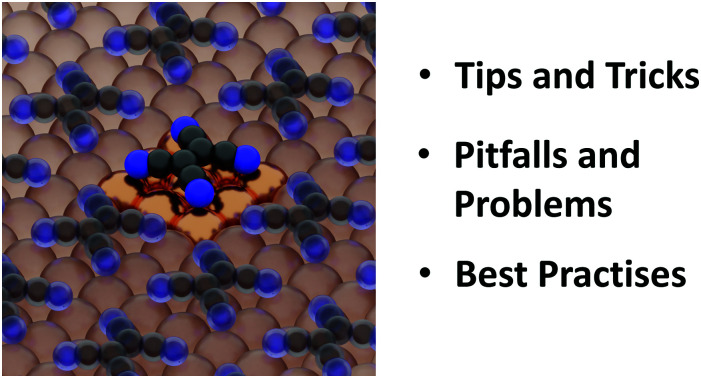

## 1 Introduction

Interfaces between organic and inorganic materials are special for a variety of reasons. From a technological point of view, inorganic materials and organic molecules exhibit complementary properties: For instance, inorganic materials tend to have higher charge-carrier mobilities, while organic materials exhibit stronger light-matter coupling and are easier to modify chemically.^1–3^ Organic molecules may, for instance, be tuned to obtain a specific band gap for light absorption or emission. Moreover, hybrid inorganic–organic interfaces determine the functionality of essentially all organic (opto)electronic devices,^4–6^ like OLED-TVs or AMOLED displays. For these reasons, they have been in the focus of research on organic semiconductors already for several decades.

Hybrid inorganic–organic interfaces are also highly interesting from the perspective of fundamental science, as they form a bridge between the traditional worlds of chemistry and physics, combining delocalized and localized electronic states. The strength and nature of the interactions between an inorganic and an organic system can vary substantially, ranging from purely physisorptive systems, where van der Waals interactions dominate, to strongly chemisorptive interactions, where new bonds are formed (and, inevitably, other bonds are weakened or broken).^7,8^ Accurately describing these different worlds on equal footing poses a formidable technical challenge, especially if such a description is based on first-principles computational simulations.

This challenge can be loosely separated into two main aspects: On the one hand, there is the “level of sophistication” of the computational method that needs to be sufficient to describe the relevant physics of the interface, *i.e.* the “methodological challenge”. Although both density functional theory and the solution of the Schrödinger equation using a many-electron wavefunction method are formally exact, in practice every practically applicable variant of these approaches is based on certain fundamental approximations that limit the physical effects it can capture. Such approximations comprise, *e.g.*, the choice of a specific approximate density functional in DFT approaches or using trial wave functions of limited flexibility in (post) Hartree–Fock calculations. A second, often overlooked aspect is the “numerical challenge”: This arises from the many possible numerical strategies and the specific settings for solving the system of equations resulting from the chosen electronic structure method. These options include the choice of the type of basis set, the algorithms used to find the minima for the electronic and/or geometric structure, the precision with which the minima are determined, *etc.* Here, many choices that perform well for crystalline inorganic materials (such as metals) fare much worse for molecules and *vice versa*. Most electronic structure software packages employ robust default settings that ensure convergence for a variety of systems. These settings are sometimes more geared towards crystalline inorganic materials or towards molecular systems, making the code more efficient for one material class than for the other. However, the ideal choices of parameters and algorithms become more difficult when different classes of materials are combined, which is naturally the case for hybrid inorganic–organic interfaces. In the best case, this may mean that the defaults cause the calculations to run inefficiently, so that they take longer than necessary to complete. In the worst case, applying the wrong algorithm – or the right algorithm wrongly – can render calculations numerically unstable or can lead to results that are physically incorrect.

The goal of this article is to provide a detailed account of the “methodological” as well as the “numerical/technical” challenges associated with modelling hybrid inorganic–organic interfaces. To achieve this, we will first discuss (in Section 2) in which respects the inorganic and organic components of a hybrid interface differ from a physical point of view. This provides the basis for the remainder of the discussion: in Section 3, we will briefly address model building, *i.e.* the necessary considerations when creating a model of a system that will be simulated and eventually compared to a real-world experiment. Section 4 continues by briefly discussing the advantages, disadvantages, and limitations of different popular computational methods that are used to model hybrid inorganic–organic interfaces. Due to its practical relevance, we will mainly focus on density functional theory with the different exchange–correlation approximations and van der Waals correction schemes that can be employed to model hybrid inorganic–organic interfaces. In Section 5, we will then deal with the algorithms, numerical approximations and settings employed in common software packages. Using examples, we will show, *inter alia*, how the uncritical use of numerical default settings may yield incorrect results. In addition, we will highlight the problems associated with geometry optimizations and the different optimization strategies, which can lead to significant differences in the calculated interface geometries.

During the following discussion, we will especially address questions relevant to practitioners. These include aspects regarding the general strategy employed for modelling interfaces (*e.g.*, extended interfaces with periodic boundary conditions *versus* aperiodic interface models), as well as specific details such as the optimal choice of numerical parameters, *e.g.*, for a rapid convergence of the self-consistent field algorithm or for the geometry optimization. We provide a diverse, but not exhaustive, set of examples from our experience gathered over more than a decade of interface simulations, but also show new simulations performed to illustrate certain arguments. This is done in an effort to establish a set of best practices. Wherever possible, we point out how chemical intuition or physical understanding can guide the choice of the optimal algorithms and their settings. We expect that this work will help newcomers in this field to obtain reliable results more efficiently, but we are confident that even seasoned veterans may still learn a thing or two. We definitely did when compiling this work!

## 2 Some fundamental differences between inorganic and organic materials

An inorganic–organic interface consists of two very dissimilar components: An inorganic component (“the substrate”) and an organic component (“the adsorbate”).††Note that, of course, the role of substrate and adsorbate may be reversed, *i.e.*, the substrate could be organic and the adsorbate inorganic. The substrate is either a metal, a semiconductor, or an insulator, and typically much thicker than the adsorbate, which consists of molecules adsorbed in a (sub)monolayer or as a thin film. In this section, we will briefly discuss the most salient differences in their electronic properties. These differences will accompany us through the rest of this work, as they are the reason for many of the numerical challenges the simulations face. Before we start with the discussion, it is important to note that the possible components of hybrid inorganic–organic interfaces encompass – quite literally – the whole of chemical space. To keep the discussion focused and avoid derailing the topic, we have to simplify and generalize several statements regarding the different materials classes, fully knowing that there are several exceptions and further physical differences that we cannot mention here. Overall, we highlight three important differences in electronic structure between organic and inorganic compounds:

### 2.1 Electronic states – orbitals and bands (Fig. 1a)

Electronic states in molecules are typically discussed in terms of molecular orbitals, *i.e.*, as discrete energy levels. Conversely, electronic states in crystalline inorganic materials are commonly described as continuous bands. On a formal basis, the two pictures are strictly equivalent: A band structure is just a sorting of orbitals (which, in a periodic structure, are infinitely extended) using the associated wave vectors (which can be obtained, *e.g.*, by a Fourier analysis). The band structure picture can be used to reveal a major difference between two materials classes: Metals and semiconductors (and, more rarely, also insulators) show appreciable band dispersion, *i.e.*, a pronounced dependence of the energy of the electronic states on the wavevector. The reason for that is that the individual building blocks (which are often just one or a few atoms) interact with each other over multiple unit cells. Conversely, in organic materials the wave-function overlap between neighboring molecules is typically much smaller, leading to bands with comparably smaller band dispersions. In fact, even in high-mobility materials like rubrene, band dispersions in π-stacking direction amount to only a few tenths of an eV.^9^

**Fig. 1:**
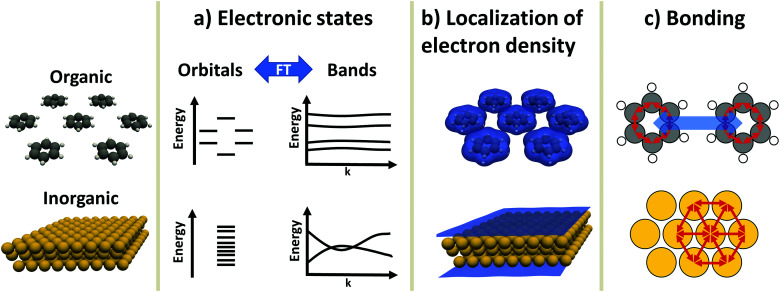
Differences between organic and inorganic materials. Shown is a monolayer of benzene and a metal slab (here, Au) as prototypical organic and inorganic component, respectively. (a) Schematic depiction of the electronic states in the orbital picture and the band picture. Due to the interaction between the atoms, the metal exhibits many energetically similar states that form (dispersing) bands due to the interaction between the atoms. Conversely, each benzene molecule has several molecular orbitals, which hardly interact with each other, giving rise to (almost) flat bands. (b) Electron density, as calculated with the PBE functional, shown at an isovalue of 0.0025 e bohr^−3^. While the metal shows an extended, rather smooth and homogeneous electron distribution, the electron density of the organic component is localized on the individual molecules and becomes small in between. (c) Schematic depiction of the bonding in the components. Each benzene molecule is held together by directed covalent bonds, while between the molecules, non-directed van der Waals interactions dominate the cohesion. The metal is held together by metallic bonds. Like covalent bonds, these originate from wave function overlap, but they contain multiple bonding partners at the same time (which also increases their isotropy).

### 2.2 Variation of the valence electron density (Fig. 1b)

Crystals consisting of elemental metals and simple semiconductors typically have a relatively uniform, weakly varying valence electron density. Conceptually, their electron densities can, thus, be reasonably well described using models derived from the homogeneous electron gas, or weak perturbations to it (like the local density approximation or the generalized gradient approximations within density functional theory). This contrasts with organic molecules, where the charge densities in the regions between the individual molecules can drop by orders of magnitude, resulting in much larger gradients of the electron density. Hence, for organic materials hybrid functionals tend to yield more accurate properties than (semi-)local functionals. This situation is primarily a consequence of interatomic distances between atoms of different molecules being significantly larger than interatomic distances of neighboring atoms within the same molecule. Additionally, the periodic repeat units of the inorganic crystals relevant in the present context are typically small, encompassing only one or a few atoms, resulting in lattice vectors of only a few Å in length. Due to the size of molecules, the unit cells of molecular crystals or adsorbates are typically much larger than those of a metal or semiconductor substrate such that the above-described charge density variations occur on length-scales much larger than the size of the unit cell of the substrate.

A further reason for qualitatively different charge distributions in organic and inorganic materials is that metals and many (conventional) semiconductors exhibit only non-polar (or very weakly polar) bonds. In contrast, organic molecules can contain highly polar bonds (such as O–H, N–H, C=O, *etc.*).

### 2.3 Chemical bonding (Fig. 1c)

Metals and most insulators have high coordination numbers, since they are held together by isotropic forces (*i.e.*, metallic and ionic bonds). Semiconductors and organic molecules are primarily held together by covalent forces, which are strongly anisotropic. Conversely, van der Waals forces, which for most organic molecules are both the major source of cohesion in molecular crystals and a major factor in the interaction between substrate and adsorbate,^7^ are (to a first approximation) isotropic.‡‡Note that, technically, van der Waals forces are anisotropic as a consequence of the anisotropy of the polarizability of the system. However, this anisotropy is only weak and mostly ignored in modelling approaches.

These three major differences between organic and inorganic systems each require special consideration when performing first-principles calculations: For instance, the localization of states and the variation of the electron density influences the applicability of electronic structure methods and basis sets used to describe the components (see Section 3). This poses a sizable challenge when combining differently bonded systems at a hybrid interface. Moreover, the different nature and directionality of the above-mentioned forces complicates the structure optimization, as will be illustrated in Section 4. A further challenge in modelling periodic hybrid inorganic–organic interfaces is the requirement of describing organic and inorganic components in the same unit cell. This requirement of commensurability often leads to very large super-cells, where a large number of substrate atoms need to be accounted for.

Finally, it should be emphasized that the nature of the interaction between inorganic substrates and organic adsorbates strongly depends on the considered interface.^4–6,8,10^ The interaction can perturb electronic states only weakly, being almost exclusively driven by van der Waals interactions, or it can be very strong and include the formation of covalent bonds.^7,11^ Additionally, charge is often transferred across hybrid inorganic–organic interfaces.^4–6,8,10^ At interfaces, the presence of dipoles can also play a different role than for bulk materials or isolated molecules,^10^ impacting charge- and energy-transfer across the interface,^4,12^ modifying the lateral charge-distribution at the interface,^13^ and reshaping molecular states.^14^ Regular assemblies of dipoles also massively impact the electronic structure of interfaces through so-called “collective electrostatic effects”.^4,8,10,15^

## 3 The structural model

The first step of any first-principles calculation is to set up a structural representation of the system to be modelled. Realistic systems extend over mesoscopic length-scales, typically containing too many atoms to be explicitly considered in a quantum-mechanical simulation. Furthermore, only in very few cases the atomistic structure is known *a priori* in full detail. Therefore, an (idealized) abstraction of the real-world situation must be used. The choices made in this abstraction are crucial, as they will determine the outcome of the computation, potentially resulting in fundamental deviations from the actual situation. Even if a perfect simulation method was available, it could not yield sensible results if the structural representation oversimplifies reality. It is, therefore, imperative that the structural model contains all the physical and chemical aspects that are relevant for the considered real-world system. Thus, in this section, we will review several common approaches used to set up models for hybrid inorganic–organic interfaces and discuss their impact on the outcome of the calculations.

**Fig. 2:**
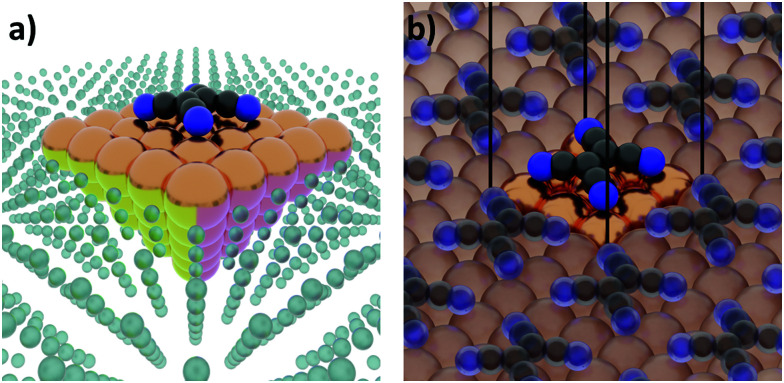
Schematic representation of tetracyanoethylene (TCNE) on a fcc(100) surface in (a) a cluster calculation and (b) with periodic boundary conditions.

Interface calculations can be separated into two major categories, depending on the spatial extent of the chosen model system. In an open boundary or “cluster” calculation, illustrated in Fig. 2a, a finite-size cluster is cut from the actual, extended interface. It is then modelled as a finite system, either on its own or “embedded” into a surrounding medium that is then typically treated at a lower level of theory. Alternatively, a slab type calculation can be performed. It employs periodic boundary conditions to infinitely repeat a central unit cell, as illustrated in Fig. 2b. Both open and periodic boundary calculations have their merits. Which approach is preferable depends on the system and the scientific question that should be addressed. At the same time, both approaches also face their own (notably different) challenges, as will be discussed in the following.

### 3.1 Cluster models of hybrid inorganic–organic interfaces

The vast majority of studies on extended hybrid inorganic–organic interfaces use periodic boundary conditions. However, finite cluster models of interfaces are well-suited for interface problems where the process or property to model is spatially localized, exhibits quantum confinement, or only affects a single molecule.^16^ These could be, for example, interfaces formed by nanoconfined systems such as nanoclusters or small nanoparticles. For extended interfaces, cluster models can be preferable when, *e.g.*, the modelled coverage is extremely low or when there is only an individual molecule on a surface, which is often the case in catalysis-related problems. A similar situation is encountered in single molecule junctions, *e.g.*, in break-junction experiments, where one typically aims at a situation in which only a single molecule forms the bridge between two electrodes.^17^ Modelling a system as “non-periodic” is also the method of choice when dealing with situations where the objects of interest are at such large distances that they do not significantly influence each other. This, for example, applies to adsorbates at moderate coverage in the absence of island formation, as well as to surface defects (again, for relatively low coverage). For such systems the unit cells in periodic boundary conditions would have to be made enormously big to prevent effects associated with the limited unit cell size, which introduce spurious interactions (especially if the defects or adsorbate molecules are charged).^18–21^ In cluster calculations such spurious interactions cannot exist by design.

Nowadays it is possible to deal with clusters containing hundreds^22,23^ or even thousands^24,25^ of atoms. However, sometimes there are benefits to creating relatively small cluster models containing “only” a few tens of atoms. This allows to study interface properties with high-level methods, including highly correlated wave-function-based methods or many-body perturbation theory.^26,27^ For certain spectroscopic observables, this arguably yields more accurate properties than when relying solely on DFT.^28^ A further decisive advantage is that the calculated systems do not need to be charge-neutral (in contrast to periodic boundary calculations, as will be discussed below). This is particularly useful when considering, *e.g.*, charged defects and core-level excitations, or when calculating ionization energies/electron affinities from the energy difference between a charged and an uncharged cluster (ΔSCF-approach).^29,30^

There are two main challenges associated with creating a cluster representation: One has to decide which part of the substrate is cut out, *i.e.* the size and the shape of the cluster. Additionally, one has to decide how the fringe of the cluster is treated, *i.e.* whether (or how) it needs to be saturated or embedded into a realistic environment in order to avoid artefacts. A further obvious problem when using small substrate clusters are so-called finite-size effects, where the spatial confinement of the wavefunction in the cluster increases the band gap.^29,31^ For semiconductors, this leads to quantitatively too large band gaps. The situation is, however, more problematic for metal clusters, which form a finite band gap (and are, thus, not metallic at all).^29,32^ Particularly problematic in this context is that electronic and optical properties of metal clusters converge very slowly with cluster size.^33^ For some properties, this problem can, in principle, be mitigated by using very large clusters or using appropriate extrapolation techniques.^34^ However, such large clusters are often computationally unfeasible. A second, related problem is that the interaction between substrate and adsorbate can change non-trivially with the size and shape of the cluster.^35,36^ Interestingly, this problem appears to be less serious when dealing with large, π-conjugated, flat lying molecules.^29^ Another issue is that clusters cut out from the bulk structure (say, an fcc crystal structure of a coinage metal) are often not stable in this form.^37^ This can outright prevent a geometry optimization of the substrate, since the cluster would disintegrate. In practice, this is sometimes solved by constraining the positions of the substrate atoms,^37^ which, however, impedes a comprehensive determination of adsorbate geometries, since surface relaxations can be accounted for only to a limited degree.

Furthermore, cutting a cluster from a substrate creates additional surfaces at its rim. For semiconducting substrates, this requires “breaking” several covalent bonds. The remaining “dangling bonds” often give rise to states in the band gap, which artificially increase the reactivity of the cluster. To obtain reliable results, it is, thus, necessary to remove these dangling bonds. This can be done either by artificially saturating them, *e.g.* with (pseudo)hydrogen atoms,^38–44^ or by employing density embedding methods.^16,31,45–50^ In the latter case, the cluster, which is computed using an expensive method, is embedded into a surrounding material that is significantly more extended, but described by a computationally less demanding approach (often, specially designed pseudopotentials). Several density embedding schemes have been developed for metal clusters,^50,51^ which are mostly focused on predicting adsorption energies and excited-state properties.

As mentioned above, it is important to be aware that most cluster models focus on only a single (or at most a few) individual molecules adsorbed on the surface. They are, therefore, generally not well-suited for mapping a situation in which inter-molecular interactions are relevant or even dominant. This is, for example, the case when the adsorbate structure is a direct consequence of adsorption-induced surface reconstructions^52–58^ or intermolecular interactions (as for upright-standing molecules in ordered self-assembled monolayers, SAMs).^59–62^ Such SAMs typically consist of long aliphatic or aromatic backbones, which are chemically bonded to the substrate *via* docking groups. In these systems, the chemical docking of a single molecule to the substrate might be properly described by a cluster model, but to obtain reliable electronic properties of the interface, also suitable embedding schemes need to be applied.^63–65^ At the same time, these systems also have sizable inter-molecular interactions between molecules, which are mostly determined by electrostatic and van der Waals forces.^66^ Removing the surrounding molecules in a cluster model also removes these interactions. As a result, rather than remaining upright, as they would be in a real-world sample, in a simulation the isolated molecules would fall over, which fundamentally changes the physical properties of the interface (see also Section 5). Even for flat-lying molecules, neglecting the interactions with neighboring molecules in a cluster calculation can be particularly problematic, if their electronic nature changes due to intermolecular interactions. This is, for example, the case for dyes such as indigo, where the formation of hydrogen bonds triggers an extension of the aromatic π-system.^67^ Notably, even if the size of the cluster is extended to accommodate several interacting molecules, edge effects would likely be unavoidable.

Besides the two rather specific examples of self-assembled monolayers and H-bonded dyes, cluster calculations, by design, miss so-called collective (also termed cooperative) electrostatic effects.^68–74^ This term refers to the fact that extended sheets of dipoles, which are commonly encountered at interfaces, affect the electrostatic potential landscape in a way that is fundamentally different from the impact of an isolated dipole.^10,75^ This directly affects the energies of the electronic levels of the materials and has a strong impact on several observables, such as X-ray photoemission spectra (*i.e.*, core-level energies)^76^ or ballistic electron transport through molecules,^77^ as well as on the overall magnitude of the charge transfer.^78^ In principle, these collective effects could be modelled in a cluster approach by using a sufficiently large cluster containing multiple molecules, but whenever an extended sheet of dipolar entities is replaced by a finite-size cluster, massive electrostatic edge-effects occur.^70,79^ Thus, to date the only strategy for correctly capturing collective electrostatics in conjunction with a cluster approach appears to be the use of suitable embedding schemes.^65^

### 3.2 An overview of the repeated slab approach

**Fig. 3:**
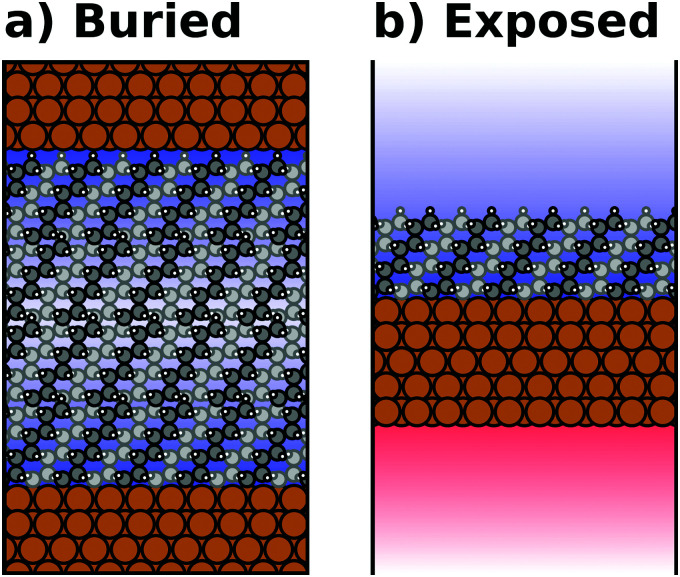
Schematic representations of (a) a buried interface and (b) an exposed interface, consisting of alkyl chains on a metal slab. The background gradients indicate the electrostatic potential.

Most calculations for hybrid inorganic–organic interfaces are, arguably, performed using periodic boundary conditions as indicated in Fig. 2b. Therefore, the peculiarities of this approach shall be discussed in more detail here. There are two subclasses of this ansatz, “buried interfaces” and “exposed interfaces”. As shown in Fig. 3a, in a “buried” interface, the whole unit cell is filled with inorganic and organic material. Due to the periodic boundary conditions in the direction perpendicular to the buried interface, there are two separate interfaces between the organic and inorganic material, which may not be identical.

Buried interfaces are the model of choice for the simulation of hybrid inorganic–organic interfaces with thick adsorbate films. This includes, for example, multilayer adsorption of small molecules or truly three-dimensional heterostructures.^80^ Conversely, “exposed” interfaces are the model of choice when the simulations are connected to surface-science experiments of solid/vacuum and solid/gas interfaces. While most issues that we will discuss below pertain to buried as well as exposed interfaces, the focus in the following will be on exposed interfaces due to their prevalence in literature.

Exposed interface calculations require the inclusion of a region of vacuum, as shown in Fig. 3b. The purpose of this vacuum region is to decouple the periodic replicas perpendicular to the interface, which are generated as a consequence of the employed boundary conditions (see below). The semi-infinite substrate is modelled by a finite number of atomic layers and the exposed interface(s) frequently contain only a (sub)monolayer of the adsorbate, usually only on one side of the metal slab. In the past, adsorbate layers have sometimes been placed on both sides of the substrate to prevent spurious polarizations. This approach is now outdated, as alternative approaches to account for an asymmetry of the slab have been implemented in the majority of the commonly applied codes, as discussed below.

Most band structure codes employ periodic boundary conditions in all three dimensions. As the buried and exposed interfaces are intrinsically only periodic in two dimensions (unless the buried interface is meant to model a periodic organic–inorganic heterostructure), in the simulations there is also the above-mentioned periodic repetition of the substrate(s) and the adsorbate(s) in the direction perpendicular to the interface. In conjunction with exposed interfaces, this strategy is commonly referred to as the “repeated slab approach”.

Despite their name, not only cluster, but also periodic calculations may suffer from various finite size effects:

• When the cell contains too little vacuum, the periodic replicas in *z*-direction may interact with each other. Provided that the vacuum is large enough to prevent wave-function overlap (approx. 15–20 Å), this interaction is not of quantum-mechanical nature. Rather, electrostatic interactions and polarization effects play a role, as will be discussed in detail in the next section.

• Unit cells can be laterally too small to represent the actual experimental situation. This can be the case, for example, when a low-coverage situation should be modelled, but the size of the unit cell in the computation needs to be restricted due to computational limitations. Too tight packing of adsorbates then results in spurious interactions between lateral replicas, which can lead, *e.g.*, to geometrical distortions of the adsorbate molecules or to inaccurate adsorption energies, especially when encountering spurious electrostatic interactions between charged adsorbates.

• Finite-size effects can also occur when the number of atomic layers chosen to represent a semi-infinite substrate is too small. As in the above-discussed case of cluster calculations, this can result in an artificial band gap and/or artificial surface states of the substrate. In contrast to clusters, where no clear construction recipe exists to eliminate artifacts due to such finite-size effects, for slab calculations the thickness of the substrate can be systematically increased by adding further layers (using the bulk crystal structure of the substrate) until these finite size effects disappear.

An advantage of periodic boundary conditions is that substrate slabs are usually stable structures. It is, thus, both possible and sensible to relax the geometry of the substrate when it comes into contact with the adsorbate. Often, these geometry optimizations lead to partial extractions of surface atoms or small-scale surface reconstructions.^81,82^ This can notably improve the stability of the interface, leading to a substantially larger (more exothermic) adsorption energy. It is common practice not to relax the complete slab. Rather, only the layers closest to the adsorbate are allowed to rearrange, while the other atoms are constrained to their bulk position. The reasoning behind that strategy is that the layers away from the interface are meant to represent the bulk of the substrate. In fact, if all metal layers were allowed to relax, the atoms at the surface of the slab not covered by an adsorbate would adopt the geometry of a clean metal surface, rather than that of the bulk atoms. In this way one would model adsorption on a free-standing metal film rather than on an exposed surface. As a rule of thumb, half or more of the layers present in the slab should be kept constrained. In Section 5 we will showcase, how such seemingly unimportant “details” (like fixing the positions of all slab atoms completely) can critically influence the resulting adsorption geometry.

### 3.3 A detailed discussion of the repeated slab approach

There are additional consequences of employing periodic boundary conditions, as they imply also full translational periodicity of all observables (including the electrostatic potential, the electron density, and, of course, the ionic positions). This imposes some constraints and limitations on the simulations, which are discussed in the following paragraphs.

#### 3.3.1 Determining interface dipoles

**Fig. 4:**
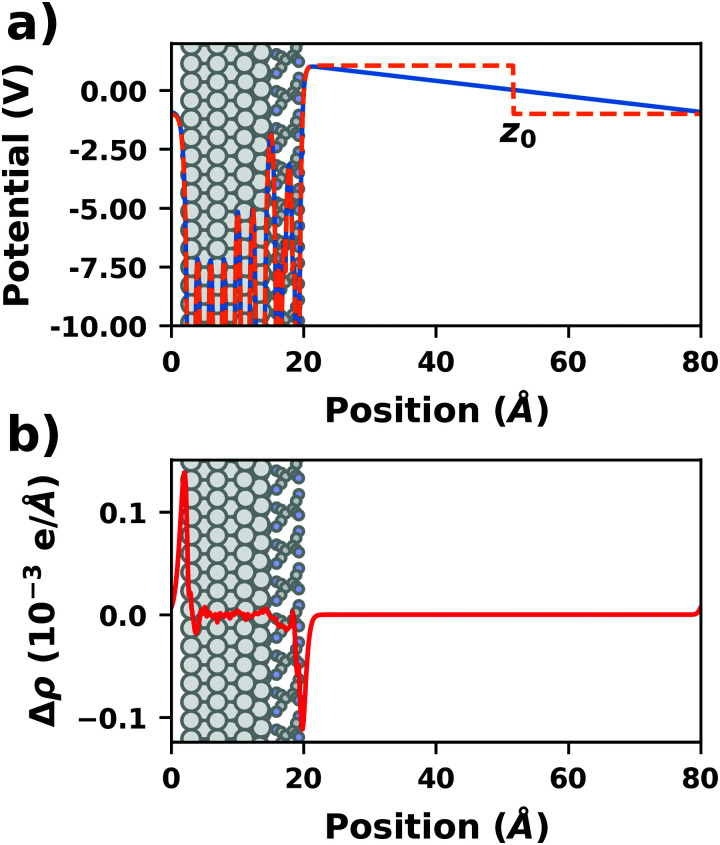
Tetracyanoethylene on Ag(111) using a unit cell with 80 Å in *z*-direction. (a) Evolution of the (plane-averaged) electrostatic potential without (blue) and with (orange, dashed) dipole correction. (b) Polarization induced by the spurious field, calculated as the difference of the electron density with and without dipole correction. The Ag/TCNE system extends from 0 to ∼20 Å, with 60 Å of vacuum above it. The data were obtained with FHI-aims using the PBE+vdW^surf^ method and a 3 × 3 × 1 *Γ*-centered *k*-grid. The original calculations can be found at http://dx.doi.org/10.17172/NOMAD/2019.10.16-1.

Periodic boundary conditions require that the electrostatic potential must have the periodicity defined by the unit cell. Therefore, the potential must not change for translations by (multiples of) a unit cell vector. In particular, the electrostatic potential must be identical at opposite edges of a unit cell. In the example shown in Fig. 4a, which displays the electrostatic potential of a tetracyanoethylene (TCNE) monolayer adsorbed on Ag(111), this requires identical values of the electrostatic potential at 0 and 80 Å (the two limits of the unit cell in the direction perpendicular to the surface). Thus, there can be no net dipole moment within a unit cell. In practice, when calculating a periodic structure that would have a dipole moment, *e.g.*, because of charge rearrangements at the interface or due to polar adsorbates, an electric field appears that generates a potential gradient in the unit cell such that the potential shift induced by the system's dipole moment is exactly cancelled. This is shown by the blue curve in Fig. 4a. The magnitude of the artificial electric field depends linearly on the magnitude of the dipole density (*i.e.*, dipoles per surface area of unit cell) and inversely on the thickness of the vacuum region. A small vacuum region therefore leads to a large field, a large vacuum to a small field. Note that this field is not a feature specifically implemented into certain program packages. Rather, it occurs automatically because of the periodic boundary conditions. The spurious field is most clearly visible in the vacuum region separating two periodic replicas of the slab. Intimately linked to that field is an artificial polarization of the slab. This artificial polarization is visualized in Fig. 4b, which shows the plane-integrated difference between the electron densities of the TCNE/Ag(111) interface with and without the spurious field. Interestingly, although the metal is much more polarizable than the TCNE molecule, the polarization leads to notable (if relatively small) charge rearrangements between the ends of the entire slab, *i.e.* from the top of the TCNE monolayer to the bottom of the metal slab.

The spurious field also affects the total energy of the system. A first estimate of that contribution (neglecting the polarization of the metal and the adsorbate) can be derived from the interaction between the dipole of the interface and a homogenous field.^83,84^ As pointed out by Bengtsson^84^ (because the field is internal, not external), it is given by *E* = 1/2 × *μ* × *F*, where *μ* is the dipole and *F* the field within the unit cell. In most cases, this energy contribution is small but non-negligible and, thus, might modify the calculated relative stabilities of specific configurations. The magnitude of the effect shall be illustrated by the following, example: Let us consider a dipole of 1 e Å (approx. 5 Debye), which is packed at such a density (here, *ca.* 180 Å^2^) that the resulting step in the electrostatic potential amounts to 1 V. Using a unit cell height of 40 Å, the total energy contribution from the dipole in the field is 1/2 × 1 e Å × 1 V/40 Å = 0.01 eV. Although that value is small, it is in the same range as the typical energy differences between different polymorphs of organic molecules.^85^ Therefore, it is important to never compare energies between different structures when the spurious field is not compensated. This is, for example, sometimes done for time-consuming geometry optimizations, since the correction schemes discussed below can slow down the SCF convergence.

**Fig. 5:**
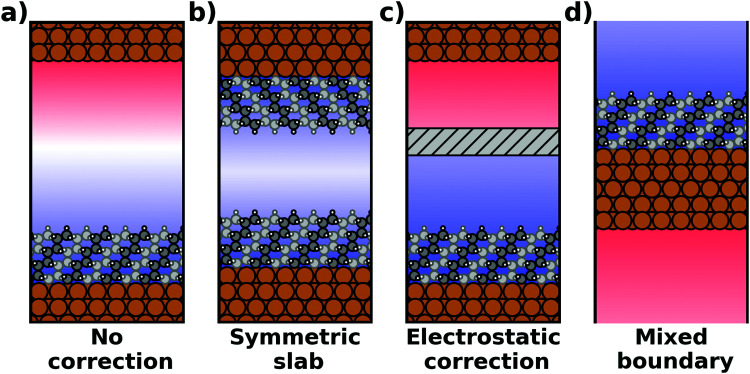
Schematic visualization of the electrostatic situation of exposed interfaces: (a) no electrostatic correction (b) a symmetric construction to prevent the formation of a net dipole in the unit cell, (c) an electrostatic correction scheme (*i.e.*, either the dipole correction or a Coulomb cutoff) and (d) a truly 2D-periodic interface with mixed boundary conditions (periodic parallel to the interface, open boundary conditions perpendicular to it). The color in the background schematically indicates the evolution of the electrostatic potential.

The spurious field and its ensuing artefacts can be avoided in several ways, which are graphically depicted in Fig. 5:

• Size of the vacuum region: in principle, one possible approach would be to increase the vacuum region until the field essentially vanishes. Unfortunately, increasing the vacuum size also increases the computational cost, especially when working with a plane-wave basis set (for a discussion on basis sets, see Section 4). Moreover, since the field decays with one over thickness, the convergence is very slow, making this not a good approach. Moreover, in this approach a surface vacuum level never forms, making an accurate determination of properties like the work function impossible. Thus, this strategy is not advisable at all.

• Symmetric slab: a second approach is to construct symmetric model systems. There, the interface is mirrored perpendicular to the *xy* plane (*i.e.*, the adsorbate is added on the bottom side of the substrate, too), causing the net potential step across the overall slab to vanish for symmetry reasons. This approach has two major disadvantages. To avoid the two sides interacting with each other, both the substrate and the adsorbate must be duplicated. Therefore, the system must be at least twice as large as for an interface with an adsorbate layer bonded to only one side of the slab. Furthermore, care must be taken to construct truly symmetric systems. This is not always straightforward. For example, the (111) surfaces of fcc-metals are stacked in an ABC manner, making it impossible to generate strictly symmetric slabs. In order to at least have identical surfaces (terminating, *e.g.*, with “A”-type layers), it is necessary to use 3*n* + 1 layers (*n* being an integer number), *i.e.*, four layers (ABCA), seven layers (ABCABCA), ten, *etc.* For other types of materials, like, for example the polar surfaces of wurtzite-type materials including the popular ZnO, (close to) symmetric slabs cannot be constructed at all.

• Dipole correction: the most common way to deal with the spurious polarization due to periodic boundary conditions is to introduce a discontinuity in the electrostatic potential within the vacuum region.^83,84^ This is also frequently referred to as the “dipole correction”. Since it is by far the most common approach used in practice, we discuss it in more detail in the following: the first step is to define the position of the plane (parallel to the interface) where the discontinuity of the electrostatic potential is created. In Fig. 4, we designate this position as *z*_0_. For all practical intents and purposes, this position separates the unit cell into a region that is “below” and a region that is “above” the slab (the red and blue regions in Fig. 5c). The choice of the position is, to some extent, arbitrary, but it should be far away from the electron density of the slab to ensure that lateral variations in the potential due to the finite extent of the adsorbate molecules have decayed. While *z*_0_ can be chosen manually, today many codes determine this position automatically, choosing either the center of the vacuum or the position in the unit cell where the electron density is minimal (for numerical reasons, plane-wave basis sets exhibit very small but finite electron density in the vacuum region). The next step is to determine the magnitude of the potential discontinuity that is necessary to compensate for the asymmetry of the slab. The specific implementation differs between different code packages, but there are two main strategies how this can be done: Either, (1) the dipole moment of the unit cell in *z* direction is explicitly calculated by integrating over the plane-averaged charge density (electrons and nuclei) between *z*_0_ and *z*_0_ + *a*_3_ (where *a*_3_ is the length of the lattice vector perpendicular to the surface). Alternatively, (2) rather than the dipole moment, the gradient of the electrostatic potential at *z*_0_ (*i.e.*, the electrostatic field at this position) is numerically calculated, and a field with opposite polarity is applied by introducing the appropriate step in the potential. Either method needs to be repeated self-consistently throughout the SCF cycle until the system's dipole is fully compensated by the correction. Although this procedure generally increases the number of cycles required to reach SCF convergence, the additional computational cost tends to be relatively small, especially compared to the other strategies described before.

When applying the dipole correction in practice, some technicalities should be considered. First, the approach requires that *z*_0_ is in a region where there is no electron density. Otherwise, especially for unit cells with a large vacuum region, moving *z*_0_ by a small distance can result in significantly different dipole moments when using method (1). Similarly, also method (2) will fail, since the potential gradient differs for different values of *z*_0_ if there is non-vanishing electron density in the vacuum region. The second technicality to keep in mind is that both procedures only work if the potential at *z*_0_ is spatially homogenous, *i.e.* if there are no significant variations in the potential within the *xy*-plane. To achieve this, *z*_0_ needs to be placed sufficiently far away from the interface. Due to collective effects, the field emanating from the dipoles decays very quickly.^68^ Therefore, choosing *z*_0_ at a distance from any atom in the slab that is larger than the distance between the dipoles on the surface can already be sufficient. Consequently, to be able to find a suitable position for *z*_0_ in cases where there is only one dipole (*i.e.*, typically one adsorbate molecule) per unit cell, the height of the vacuum region should be in the same range as (and ideally larger than) the lateral extent of the unit cell. For large supercells, this provides the limiting factor for how small the vacuum region can be chosen in a slab calculation.

• Other approaches: there are many more approaches to achieve a similar electrostatic decoupling of periodic replicas: for example, the long-range electrostatic interactions across images perpendicular to the surface can simply be truncated in Fourier space. This so-called Coulomb cutoff method is fast, simple to implement^86^ and effectively equivalent to the dipole correction approach.^87^ Other approaches include the minimum image convention, where the Coulomb operator is still periodic, but contributions from neighboring unit cells are removed.^88,89^ Yet another approach to eliminate spurious electrostatic interactions across images is the use of open boundary conditions perpendicular to the surface. Then, the system is periodically repeated in *x* and *y* directions, while it is non-periodic perpendicular to the surface (mixed boundary conditions). This can be achieved by solving the electrostatic potential on a grid and imposing reflecting (Dirichlet) conditions when solving the Poisson equation, which is used to evaluate the Hartree potential.^90,91^ All these methods display a slightly different behavior^92^ and are occasionally used for more non-standard situations that involve complex boundary conditions or electrostatic environments.^93^

Periodic boundary conditions directly imply that a sample is infinitely extended. While this is obviously not the case perpendicular to the surface, it is also an approximation parallel to the surface, since every real-world sample is finite in size. One consequence of this is that, if an adsorbate has a dipole moment parallel to the surface, it vanishes when employing periodic boundary conditions. For metallic substrates, this is less of an issue, since any dipole moment is fully screened by the metal. On non-metallic substrates, the screening is incomplete and in the actual sample lateral potential gradients prevail, which in the simulations would be eliminated by the periodic boundary conditions. However, because in practice the potential gradients are typically very small (and correlate inversely with the domain size),^94^ this artefact is typically of little relevance (unless observables like piezoelectricity are of interest).

This brings up another problem associated with employing the repeated slab approach: naturally, this approach is not suited to describe interfaces that do not display periodicity in a two-dimensional plane. One of the reasons for that can be the lack of commensurability between substrate and adsorbate, an aspect that will be discussed below. Another reason is that one might not be dealing with a flat substrate or with a homogeneous adsorbate layer, as would be the case for nanoparticles with adsorbed molecules or for single molecule junctions. In such cases, problems associated with collective electrostatics (*i.e.* steps in the electrostatic energy due to periodic arrangements of dipoles) come into play.^4,8,10^ As discussed above, cluster calculations without electrostatic embedding will fail to account for these effects of extended polar surfaces. Conversely, the periodic boundary conditions in repeated slab simulations automatically include them. This, however, becomes a problem, when the investigated system does not contain a periodic arrangement of dipoles. This, for example, applies to single-molecule junctions. Their correct description employing periodic boundary conditions requires large unit cells to prevent spurious interactions between neighboring dipoles and to avoid collective electrostatic effects in the simulations that do not occur in the actual junctions.^12,77^ Similar considerations apply when modelling local excitation processes at a surface, as in the case of final-state simulations of core-level binding energies. With periodic boundary conditions, they artificially model a core hole in each unit cell, in sharp contrast to the actual situation encountered in the experiments.^21^ In such cases, where a periodicity of the interface is assumed that does not exist in the actual system, repeated slab calculations can produce even qualitatively wrong results.

#### 3.3.2 Charged unit cells

Another issue that arises due to periodic boundary conditions is the problem of describing charged moieties. Non-neutral entities arise in several physical problems, *e.g.*, when dealing with defects in semiconductors^18,31^ or when modelling ionization processes.^95^ Within the repeated slab approach, it is generally not possible to charge only a single unit cell. Rather, periodic boundary conditions imply that all unit cells in the system carry a charge. A periodic arrangement of (non-compensated) charges, however, results in a diverging energy. This prevents convergence of the SCF algorithm for charged unit cells.

In practice, by default all band structure codes compensate unit cells containing an excess (or a deficiency) of electrons by assuming a homogenous background charge with opposite polarity, which exactly compensates the excess charge. This is a feature of the Ewald summation, which neglects the “*G* = 0 term”. Including that term would cause numerical problems and neglecting it has no consequences for neutral unit cells, as for those it is exactly zero. For charged unit cells, neglecting the “*G* = 0 term” corresponds to restoring charge neutrality by the above-mentioned homogeneous background charge. Therefore, analogous to the electric field in dipolar cells, this homogenous background charge is not a particular feature of a specific code (which could be switched on or off *via* a keyword), but rather appears automatically.

**Fig. 6:**
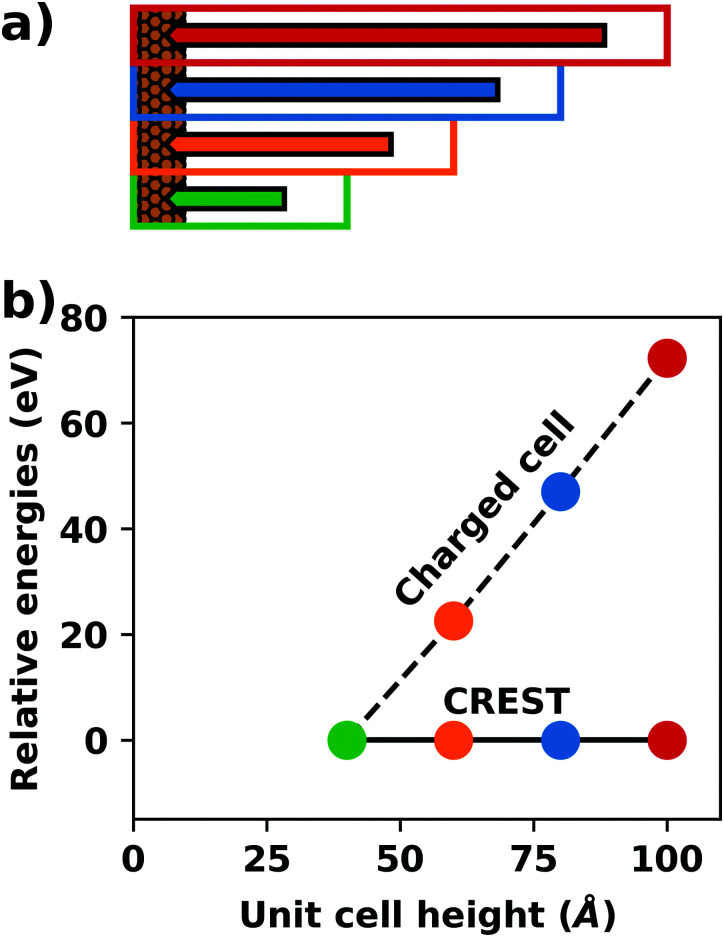
(a) Schematic representation of the slab and the evolution of the dipole when varying the unit cell height for a charged unit cell. Provided that the vacuum region is much larger than the slab, the dipole of the cell amounts to *Q*·*z*_0_/2, with *Q* being the net charge of the slab and *z*_0_ referring to the unit-cell height. (b) Dependence of the energy (relative to a 40 Å thick cell) on the unit cell height of a charged Cu(111) (1 × 1) surface unit cell where one extra electron was added and compensated with a homogenous background (dashed line) or with the Charged Reservoir Electrostatic Sheet Technique CREST^102,103^ (solid line). All calculations were done using the PBE functional and 33 × 33 × 1 *k*-points and can be found under https://dx.doi.org/10.17172/NOMAD/2020.12.11-1.

A common scenario, where homogeneous charge backgrounds are used are, *e.g.*, first principles studies of charged defects in bulk systems. Although these calculations suffer strongly from a dependence on the size of the supercell (*i.e.*, the charge density of the compensating background), several efficient correction and extrapolation schemes have been developed to compensate for these problems.^96–100^ Unfortunately, they are not applicable to interfaces:^20^ A particular problem for interfaces is that there is a Coulomb interaction between the excess charge, that is localized in the slab, and the delocalized homogenous background charge. This gives rise to a significant contribution to the total energy of the system, as shown in Fig. 6b for the example of a 4-layer Cu(111) slab containing one excess charge per 1 × 1 surface unit cell. The spurious energy contribution originates from the spurious net dipole of the unit cell and, hence, scales linearly with the thickness of the vacuum region,^101^ as shown schematically in Fig. 6a.

Two classes of approaches have been developed to deal with this problem. The first corrects for the electrostatic potential of the excess surface charge by interfering with the Poisson equation that describes the electrostatic potential^104–106^ or *via a posteriori* correction schemes based on the dielectric profile of the interface.^20^ A variety of modified Poisson solvers have recently been proposed to tackle charged surface systems in the context of electrified interfaces^107^ such as the Solvated Jellium approach^108^ and the metallic boundary conditions proposed by Otani and Sugano.^104^ Both approaches neutralize the interaction between charged cells perpendicular to the substrate. Note, however, that even there the lateral interaction between charged cells persists, requiring a careful converge of the supercell size. The downside of these approaches is that they are not yet widely available.

The second class of methods ensures that the slab as a whole is charge neutral, such that no compensating background charge is generated. This is typically achieved by intentionally adding spatially localized countercharges into the system. Two representatives of this class are the virtual crystal approximation (VCA)^18,109^ and the Charge Reservoir Electrostatic Sheet Technique (CREST):^102,103^ The VCA modifies the charge of the atomic nuclei, such that the excess charge in the cell is completely accounted for. This effectively localizes the countercharge to the slab, mitigating the size divergence problem (but not completely eliminating it, since for a fixed amount of charge there is now a dependence on the substrate thickness). Hence, the VCA is commonly used to simulate doping of bulk semiconductors, where it provides a fixed number of free charge carriers per volume, rather than per area. CREST is an extension of the VCA and models the countercharges as a charged sheet, which is placed below the substrate. The additional energy contribution due to introducing that charged sheet at a specific position in the vacuum region is then corrected analytically,^103^ eliminating the size dependence. This approach can also be used to mimic the impact of band-bending and charge transfer for adsorbates on (doped) semiconductor substrates.^43,110,111^ A conceptually similar approach is the generalized dipole correction approach,^112^ which introduces a monopole sheet as a “computational electrode” and a dipole layer in the vacuum region to decouple charged periodic images to produce boundary conditions equivalent to isolated slabs.

#### 3.3.3 The commensurability conundrum

**Fig. 7:**
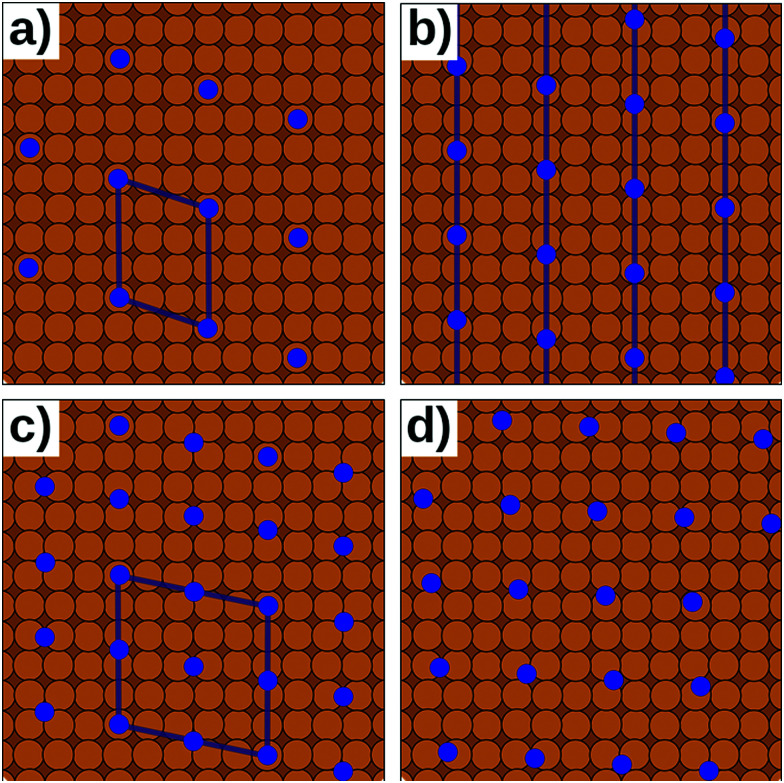
Different types of epitaxy. Orange circles represent the substrate, blue circles the adsorption site of the adsorbate. (a) Commensurate interface. (b) Point-on-line epitaxy. (c) Higher-order commensurate interface. (d) Incommensurate interface. Only a and c can be modelled with periodic boundary conditions. b and d require streching or compressing of either the substrate of the adsorbate.

Slab-type calculations can only employ a single set of periodic boundary conditions (*i.e.*, one size of the lateral unit cell). Therefore, both the substrate and the adsorbate are subject to the same lattice periodicity and must share a common supercell. This is only possible if the adsorbate unit cell is an integer multiple of the substrate unit cell (or *vice versa*) such that a common supercell can be defined,^113^ as shown in Fig. 7a and c. More precisely, the epitaxy matrix, that defines the relation between the adsorbate and substrate unit cells, must contain only integer entries. More details on how epitaxy matrices are defined and a discussion of the different types of commensurability (including epitaxy, point on line, line on line, *etc.*) can be found in a recent review by Forker *et al.*^114^ Following the Frenkel–Kontorova model,^115^ commensurate structures form only under certain conditions (coincidence notwithstanding): The interaction of the substrate with the adsorbate must be sufficiently strong such that there is a clear, energetically preferred adsorption site for each molecule. At the same time, the interaction within the molecular adsorbate layer must be sufficiently weak such that the molecules are not expelled from the potential well of that adsorption site.

In reality, of course, not all hybrid inorganic–organic interfaces are commensurate in all directions (see Fig. 7b and d). Still, when modelling an interface using periodic boundary conditions, commensurability must be enforced. If the deviation from commensurability is not too large, this can be done by compressing and/or expanding the lattice constants of either of the interface components. In practice, this implies that an appropriate supercell of the substrate and the adsorbate (probably containing 2 or more molecules) should be sought that minimize the relative lattice mismatch. When modifying the lattice constant(s), care has to be taken not to perturb the properties of the material too much, *e.g.*, by verifying that the degree to which the band structure, the work function, and similar properties are affected by the change of the unit cell parameters is still acceptable.

Even then, one should keep in mind that any distortion of the lattice parameters will make covalently bonded systems more reactive (as they are no longer in their energetic minimum). A second effect is that in a truly incommensurate system every adsorbate molecule resides at a different relative position on the substrate. Consequently, in the full layer all adsorption sites are equally sampled independent of whether they are energetically favorable or not. Conversely, when artificially enforcing commensurability, only favorable adsorption sites will be occupied. This can be most easily understood, when considering only a single molecule in a fixed supercell. Moving the modelled molecule over the substrate does not change the molecule–molecule interaction. Hence, the whole layer can be translated relative to the surface, until the most favorable molecule–adsorbate interaction is found. This and the higher reactivity of the modified sub-systems tend to lead to an overestimation of the substrate–adsorbate interaction, and, thus, to overestimated adsorption energies. However, also the opposite effect may occur, if, *e.g.*, the stretching of the substrate introduces a lattice mismatch between the docking groups of the adsorbate and the docking sites of the substrate. The magnitude of either effect can be appreciable^116^ and can easily be larger than the energetic differences between different adsorption sites or polymorphs.^117^ Consequently, for such interfaces authoritative first-principles structure search or the reliable simulation of surface phase diagrams is currently an unsurmountable challenge.

Presently, there are few approaches of which we are aware that overcome the commensurability conundrum. If the molecule–substrate interaction is very weak, certain properties can be described by modelling a freestanding monolayer defined by the periodicity of the adlayer alone, for example when simulating STM topographies. An alternative is to map the DFT interactions onto more approximate methods like interatomic potentials^116^ (*i.e.*, force fields) or semi-empirical methods. Today, this mapping can be done efficiently with the help of machine-learning methods.^118–123^ The computationally cheaper methods can then be efficiently evaluated for extensive domains containing thousands of molecules, potentially providing a more accurate representation of incommensurate interfaces.

### 3.4 Structure of the interface

The properties of an interface are strongly affected by its atomistic structure. While setting up the initial structural model of the interface (*i.e.*, the input geometry of the calculation), several assumptions need to be made that can have a fundamental impact on the obtained results. These include, *e.g.*, the size and shape of the unit cell, the number of molecules to be placed in that cell, the adsorption sites and orientations of the molecules, *etc.* Many of these decisions are immutable and will not change during the calculations. This is obvious for factors like the coverage, but also applies (at least to some extent) to aspects like the orientation of the molecules and their adsorption site, as will be discussed in the following.

The choice of the initial setup can be massively simplified when structural input from experiments is available. Unfortunately, such input is typically limited, since most experimental methods yield only incomplete atomistic insight. The immediate interface region typically comprises only a monolayer or at most a few layers of organic material, which often provides too few scattering events for X-ray diffraction to yield the full structural information. Low-energy electron diffraction (LEED) provides information about the unit cell of the organic adsorbate, but yields only limited information on the geometry of the adsorbed molecules regarding their adsorption sites or on their orientation relative to the substrate. Scanning tunneling microscopy (STM) can provide this information when performed with atomic resolution, but imaging both the substrate and the adsorbate simultaneously works only in fortunate circumstances. Also, STM can only be applied to conducting substrates. Moreover, it should be kept in mind that STM measures the local density of states, not the positions of the atoms, and that the appearance of an adsorbate in STM can be deceiving.^124–131^

Also, the composition of the substrate surface and its impact on the interface structure is often not *a priori* clear. Many metal–organic interfaces incorporate adatoms from the substrate,^8,82,132–139^ which have a decisive impact on the geometry of the organic layers as well as on the interface properties.^8,82,132,133,140,141^ A similar problem occurs for semiconductor surfaces, which can exhibit different reconstructions and surface terminations. One example for this is the ZnO(0001)-surface, which, depending on the sample history, may contain a different number of hydrogen or hydroxyl groups on the surface or form triangular pits with missing surface atoms.^39,101,103,142–144^ Like for metal surfaces, these surface modifications can substantially change the interface geometry and chemistry.^145,146^ A particular challenge in this context is that adatoms or hydroxyl group are frequently not detectable in (especially LEED or STM) experiments. Rather, their presence can often be inferred only indirectly, *e.g.*, through their impact on the geometry, which requires a combination of theoretical and experimental techniques.

In lieu of experimental information, scientific studies often consider only a single molecule in a large supercell in order to “avoid spurious interactions” or to model a theoretical low-coverage regime. This strategy does not account for the fact that the absence of interaction may be spurious in itself, or that at nominally low coverages there could be island growth, with layers that locally display dense packing. Spuriously large unit cells particularly affect the adsorbate geometry: For (mostly) upright standing molecules, the coverage and packing motif crucially determines their tilt angle,^147^ but also for simple, weakly interacting, flat-lying molecules, a dependence of the adsorption height on the molecular coverage has been reported.^148,149^ Moreover, a surprisingly large number of molecules shows coverage-dependent re-orientations, phase transitions, or conformational and chemical changes.^62,150–160^

**Fig. 8:**
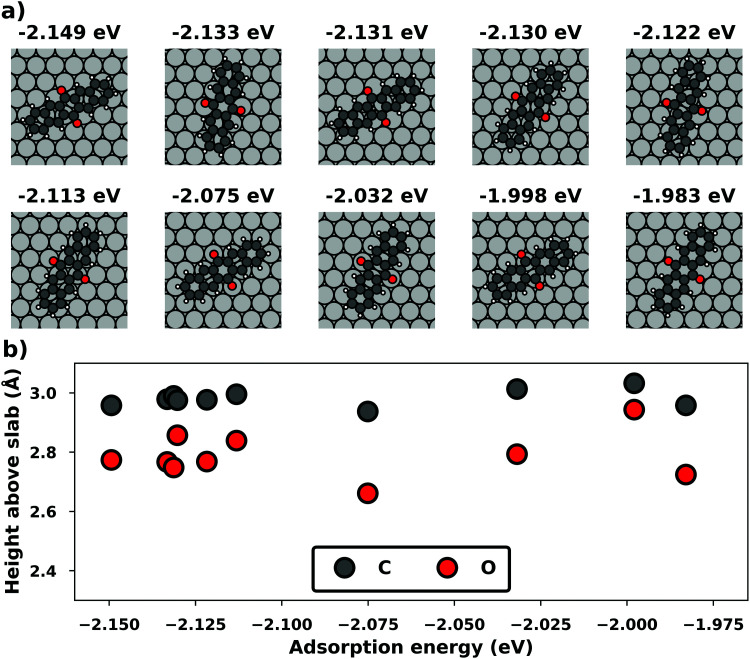
Overview of the different local minimum structures for the adsorption of pentacenequinone on Ag(111), calculated with 3 × 3 × 1 *Γ*-centered *k*-point grid using PBE+vdW^surf^. (a) Top view of the different structures, together with their adsorption energies, calculated as the energy difference between the system and the sum of slab and molecule on their own. (b) Average position of carbon (grey), and oxygen (red) atoms above the topmost silver layer, ordered according to their adsorption energy. Further calculation details can be found in Jeindl *et al.*^162^

Even if the “single molecule in a supercell” approach is the best suited model (*e.g.*, because the molecules repel each other on the surface), finding the correct geometry (*i.e.*, adsorption site, orientation, conformation, *etc.*) is not necessarily straightforward. Even for an isolated molecule, multiple possible adsorption sites and conformations exist, often with notably different properties. This point is exemplified in Fig. 8 for the case of 6,13-pentacenequinone on Ag(111). Systematically scanning the potential energy surface for a single molecule in an approx. 252 Å^2^ large supercell, we detect 10 different stable local minimum structures. The displayed data have been calculated with PBE+vdW^surf 161^ and were taken from Jeindl *et al.*^162^ The different structures and their adsorption energies are depicted in Fig. 8a. Notably, six of the ten found structures are almost isoenergetic (*i.e.*, within 50 meV); only four structures are substantially less stable. Despite the very similar adsorption energies, the adsorption heights vary substantially between the structures (especially for the oxygens), as shown in Fig. 8b.

We emphasize that the existence of a variety of different adsorption structures is not a peculiarity of this example. Even for the relatively small molecule tetracyanoethylene (TCNE), a large variety of different structures has been found on various metal surfaces both theoretically^153,158,163,164^ and experimentally^134,153^ and there are plenty of additional examples.^162,165–167^ The existence of multiple minima of the potential energy surface for even a single adsorbed molecule illustrates a fundamental problem for first-principles studies of interfaces: Unless a systematic structure search is performed, it is easy to miss the global energetic minimum, which is arguably the most relevant structure. A survey of literature indicates that systematic searches are the exception rather than the rule. (Although, admittedly, it is often not clear to which extent some search based on trial-and-error was performed, but not reported. Thus, we recommend that the details of such a search are documented more often in the published literature). Even when systematic evaluations are done, they mostly rely on creating different starting points based on physical and chemical intuition followed by local geometry relaxations, rather than employing more unbiased and systematic approaches like Basin Hopping^168^ or machine learning-based algorithms.^166^ As a further complication, local relaxations have their own challenges, as will be shown in Section 5. Yet, without any systematic search, a (more or less) random local minimum will be found. Consequently, different scientists studying identical interfaces will not necessarily find the same (local) minimum structures, which negatively impacts the reproducibility of first-principles interface studies, at least unless the full geometry is provided (*e.g.*, *via* data repositories). Moreover, the variation of adsorption geometries can introduce a notable (perceived) error bar on the calculations and significantly complicates the evaluation of the performance of the employed methodology compared to real-word experiments (see Section 4). Interestingly, recent advances in global structure optimization have finally put global structure search at hybrid interfaces including predictions of their atomistic structures^168–170^ and even an assessment of different polymorphs, within reach.^165,171–173^ In passing, the authors note that their algorithm for global structure search at interfaces (SAMPLE)^165^ is available as a python package that can be downloaded from the author's homepage (www.if.tugraz.at/hofmann). Notably, global structure search also provides an overview of the potential energy surface, which then allows to either perform Monte-Carlo simulations in order to predict the morphologies of (sub)monolayers under kinetically determined growth conditions,^171^ to determine thermodynamically stable structures and phase diagrams,^165^ or to account for the impact that defects with low formation energies have on the structure.^163^

## 4 The electronic structure method

Despite recent advances in the efficiency of correlated wave-function-based methods, their application to hybrid inorganic–organic interfaces remains expensive and is mostly restricted to small (embedded) clusters.^47^ Thus, band-structure calculations based on density functional theory remain the workhorse for first-principles studies of interfaces. There exists, however, a plethora of different exchange–correlation (xc) functionals (empirical and non-empirical) that could be applied. This raises the question which xc functional should be used in practice.

Kieron Burke, one of the pioneers of modern xc functionals, stated that “Users should stick to standard functionals [*i.e.*, PBE for materials], or explain carefully why not”.^174^ This statement certainly also holds true for interfaces, although it should be amended by stating that a van der Waals correction is almost always required. Nonetheless, there are sometimes good reasons to deviate from the standard approach. Since the performance of different electronic structure methods is frequently reviewed, also in the light of interfaces,^47,175–181^ here we will focus on a quick, rather general overview of some of the most relevant xc functionals available as well as the options to account for van der Waals interactions. Note that for the sake of brevity, some of the following statements may be (over)generalizing. For a more comprehensive discussion on how to construct and choose xc-functionals (including a benchmark of their performance) the reader is referred to the recent reviews of Burke^182^ and of Truhlar.^183^

### 4.1 Density functionals for interfaces

The core idea of density functional theory, as laid out by Hohenberg and Kohn,^184^ is that the ground state properties of a system are encoded in its electron density, *i.e.* there is a functional that directly connects the systems' electron density to its total energy. Although this greatly simplifies first-principles calculations in theory, the correct functional is still unknown. Later, Kohn and Sham demonstrated that the correct electron density can also be obtained from an ensemble of non-interacting particles.^185^ For such a system, it is straightforward to write down the equations for the kinetic and the Coulomb energy. Only the functional for exchange and correlation, for which no general form is known, must be approximated. Today, it has become customary to group the different approximations to the exchange–correlation functional into several rungs of the so-called Jacob's ladder, as coined by Perdew.^186^ Within each rung, many xc functionals exist. These are either constructed to obey certain theoretical limits and sum rules, or parameterized to reproduce the properties of specific materials that were calculated with higher-level methods. Notably, these rungs roughly group the xc functionals according to their computational cost, but ascending the rungs does not necessarily mean that also the accuracy of the results increases, as we will discuss below. It is also important to note that many developments of the last 15 to 20 years do not neatly fit into these rungs, *e.g.* self-interaction corrected functionals or van-der-Waals-inclusive functionals. A detailed review of these recent developments in materials research was recently given by Maurer *et al.*^175^

The local density approximation (LDA) constitutes the 1st rung of Perdew's ladder and is the simplest of all constructions, where the xc-functional depends only on the value of the electron density at a given point in space. Although different versions of LDA functionals exist, they are all numerical parametrizations to the exchange and correlation energies of the uniform electron gas. In practice, they differ only in the functional form and parameterization, and all yield very similar results. It is well known that LDA gives a good account of the lattice constants and band structures of simple metals, but does not perform as well for molecules and semiconductors. Nonetheless, twenty years ago, LDA calculations were used routinely for interface calculations,^29^ because they exhibit an artificial energy minimum between subsystems, even if they are not covalently or ionically bonded. This was often taken to “mimic” van der Waals interactions, although this overbinding occurs for the wrong physical reason.^176,187–189^ A nice example is the case of PTCDA on Ag(111), where LDA predicts an adsorption height in good agreement with X-ray standing wave measurements.^190^ However, for most cases of hybrid inorganic–organic interfaces the adsorption distances computed employing LDA are too low compared to experiments.^176,190–194^ Furthermore, LDA also shows a strong overbinding for intra-molecular bonds in molecules (resulting in too short inter-atomic distances^195^) and, generally, performs poorly for molecular properties.^196^ Since the computational advantage over the next higher rung (GGA, see below) is very small, and due to the unsatisfactory geometries it predicts, LDA is hardly used today for modelling interfaces.

The first improvement over LDA is to account also for the gradient of the local electron density, giving rise to the Generalized-Gradient-Approximation (GGA). Due to the inclusion of the gradient, these xc functionals are called semi-local. In contrast to LDA, many different functional forms and parameterizations are in frequent use. For inorganic bulk materials, GGA functionals provide significantly better cohesive energies and lattice constants compared to LDA.^197,198^ For interface simulations, the PW91 functional^199^ has seen frequent use, since it recovers some of the artificial binding of LDA. The PBE functional,^200^ which is a non-empirical simplification of PW91, is considered today's default for solid state physics problems and is the most widely used xc functional. Multiple modified versions of PBE exist, which involve a re-parameterization to other theoretical constraints, (rPBE^201^) to constraints specifically relevant for solids (PBEsol),^202^ or to match atomization energies (revPBE^203^). In PBE, most molecules that are purely van der Waals bonded to the surface do not stick to the surface at all^175,191^ (potential artefacts from basis set superposition errors and/or the geometry optimization algorithms notwithstanding).^204^ Indeed, PBE generally tends to underbind,^205,206^ even within molecules.^207^ Its reparameterizations rPBE and revPBE are said to improve the description of chemisorption,^203^ while PBEsol slightly overestimates the adsorption energies of chemisorbed moieties.^208^ PBE has been shown to yield excellent predictions for the electronic properties of metals, especially the work function (within the experimental uncertainties).^209^ It does, however, generally overestimate the polarizability and underestimate the dipole moment of isolated molecules.^210^ Although one would expect that this poses a problem for interfaces, when the work function is determined by polar adsorbates, it is our general experience that (provided that the experimental geometry is used) the obtained results agree rather well with the experimental values obtained in ultra-high vacuum.^82,124,150,151,191,211–216^

Meta-GGAs are the next rung of functionals. They also consider the kinetic energy density, which is equivalent to the second derivative of the electron density. This makes them the logical next step up from GGAs. Multiple variants exist, such as TPSS^217^ (and its revised version revTPSS^218^) or SCAN^219^ and its revised versions rSCAN^220^ and SCAN-L.^221^ Although the description of cohesive energies of metals as well as atomization energies of molecules is improved with these xc functionals, they are, to the best of the authors’ knowledge, hardly ever applied to investigate the adsorption of large organic molecules on metal surfaces. This may be in part because accounting for dispersion forces by *a posteriori* correction schemes (see next section) is more difficult for this class of functionals (apparently because meta-GGAs already are relatively non-local, thus, undermining the concept of these *a posteriori* corrections). Still, dispersion interactions have been incorporated *via* non-local corrections of meta-GGAs^222^ (see next section). These have been applied successfully to small, prototypical interfaces.^222,223^ Unfortunately, at present we are unaware of more extensive, systematic tests for hybrid inorganic–organic interfaces, making it too early to assess the performance of meta-GGAs in the context of this work.

**Fig. 9:**
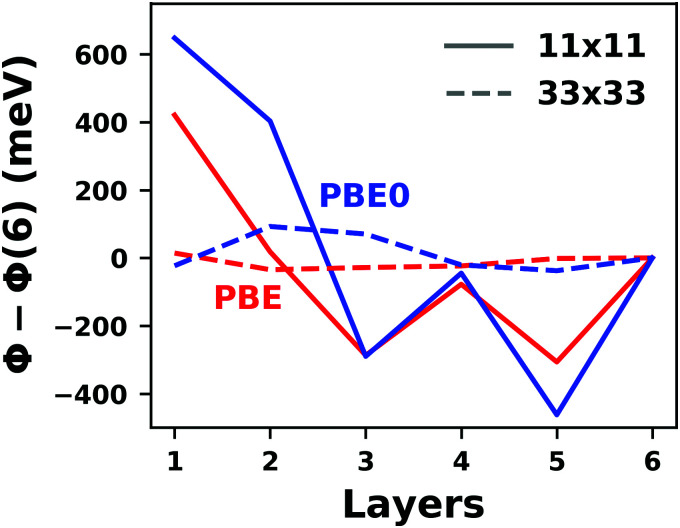
Work function *Φ* of a Cu(111) slab (relative to the work function of a 6 layer-slab) as a function of the number of layers in the slab calculated with GGA-type xc functional PBE (red) and the hybrid functional PBE0 (blue). The calculations were done for a primitive surface unit cell containing 1 atom per layer with 11 × 11 (solid line) and 33 × 33 (dashed line) *k*-points. Calculations were done with FHI-aims using the “tight” defaults. Further details and results can be found at https://dx.doi.org/10.17172/NOMAD/2020.12.07-1.

Hybrid functionals constitute the highest rung of functionals that can currently realistically be applied to hybrid inorganic–organic interfaces. In this rung, a fraction of Hartree–Fock (HF)-like exchange (sometimes referred to as exact exchange) is admixed to the complementary part of semi-local exchange. This exchange is non-local, which increases the computational effort over semi-local functionals typically by at least one order of magnitude. The computational cost is furthermore increased by the fact that hybrid functionals yield larger band dispersions, which has a two-fold impact: For metals, the larger dispersion leads to a smaller density of states (and, thus, available charge carriers) near the Fermi energy. Thus, slab-type interface calculations generally require more metal layers, *i.e.* larger systems, to provide a stable Fermi energy of the substrate, as exemplarily shown in Fig. 9 for a Cu(111) slab. Secondly, the larger dispersion generally^224^ requires a denser sampling of the reciprocal space (*i.e.*, more *k*-points) to yield converged results. Furthermore, hybrid functional-based calculations are more difficult to parallelize efficiently, which affects their scalability towards larger systems. Although these challenges are continuously addressed by new, improved algorithms, the large computational cost is an effective barrier that deters many users from routinely using hybrid functionals for interface simulations.

All hybrid functionals contain the fraction of employed Hartree–Fock-like (HF) exchange as one parameter. Similar to semi-local functionals, many different hybrid functionals with different design strategies exist. Here, only a few should be mentioned: PBE0 constitutes an extension of PBE, with a fraction of HF-exchange of 25%, as motivated on theoretical grounds.^225^ Another popular approach is the B3LYP functional, which is heavily parameterized (to molecular data) and employs 20% HF-exchange. However, B3LYP is rarely used for hybrid inorganic–organic interfaces as its molecule-focused parametrization does not provide consistent accuracy for metals or interfaces.^226^ Generally, hybrid functionals significantly improve the quality of the prediction of the properties of isolated molecules.^227^ They are, however, fundamentally problematic for metallic systems,^208,226^ because of a divergence near the Fermi-energy (a problem they share with many wave-function based methods). This problem can be mitigated by using range-separated functionals, specifically short-range hybrid functionals, such as HSE06,^228^ in which exact exchange is attenuated in the long range. This attenuation is well justified for metallic bulk systems, where exchange is effectively screened and the electron density “in the long-range” resembles a homogenous electron gas.^229^ Conversely, for systems surrounded by vacuum (such as isolated molecules), theory would require that hybrid functionals, which employ 100% HF-exchange in the long range, are applied.^230^ The latter approach is frequently used in optimally-tuned range-separated functionals,^231–234^ such as the LC-w-PBE functional.^230^ Such functionals try to solve the general band-gap problem of DFT by choosing the amount of exact exchange (and possibly the range-separation parameter) such that the IP-theorem^235^ is fulfilled, *i.e.* such that the energy of the highest occupied Kohn–Sham state is equal to the ionization energy. For interfaces, this poses a clear challenge: Individual, small molecules typically require large fractions of Hartree–Fock-like exchange to fulfill the IP theorem,^236^ while for metals, smaller values suffice.^212^ Although this problem is partly mitigated by the fact that for interacting systems, *i.e.* for molecules near a surface, the ideal amount of exchange is significantly reduced,^237^ the conundrum remains that different values would be required for the substrate and the adsorbate. A more detailed discussion of the role of exact exchange for modelling interfaces, how to potentially determine a suitable value for its relative weight, and its impact on the transfer and distribution of charges at interfaces is given by Wruss *et al.*^237^ Generally, hybrid functionals (of all kinds) tend to improve the description of molecular properties, including geometries, dipole moments and polarizabilities.^210^ Kresse *et al.* showed for pristine substrates that work functions calculated with hybrid functionals are generally smaller than those obtained from semilocal approximations.^208^ This can be ascribed to the fact that hybrid functionals yield more localized electron densities, which decreases the surface dipole of metals. Notably, the trend of smaller work functions for hybrid functionals does not necessarily transfer to the simulation of metals covered by an adsorbate layer, where the surface dipole is changed due to pushback and other effects, such as charge transfer.^212^

### 4.2 Long-ranged dispersion interactions

Van der Waals (vdW) interactions between individual atoms are quite weak, typically amounting to about 0.1 eV per atom. However, since they act between all atoms of a system, the grand total of the vdW energy for extended molecules adsorbing on surfaces or forming molecular crystals becomes quite substantial. Generally, van der Waals interactions are the main contribution to the cohesion of organic crystals^238^ and organic layers.^66^ Without them, many interfaces would be inherently unstable.^191^ Even for covalently bonded systems, a correct description of vdW interactions is crucial, *e.g.*, for the adsorption height of flat-lying molecules.^176^ In short, vdW interactions play a decisive role at hybrid inorganic–organic interfaces; maybe even so much that it is fair to say that the advent of van der Waals inclusive functionals and *a posteriori* corrections, that became commonplace for interface calculations between 2006–2010, has created a schism in the modelling of hybrid inorganic–organic interfaces. Whereas before, large deviations between experimentally and computationally determined adsorption geometries were readily accepted as a reasonable agreement, today adsorption heights within 0.1–0.2 Å of the experiment can be readily obtained^176^ and even these deviations are potentially due to numerical rather than due to physical approximations, as we show in Section 5.

The first rung of density functional theory that intrinsically includes (long range) van der Waals interactions would be the random phase approximation (RPA). Due to its large computational cost and difficulty of generating converged and numerically robust results, this method is rarely used to describe relevant hybrid inorganic–organic interfaces.^239,240^ In addition to the RPA, there are presently two conceptually different approaches that allow treating van der Waals interactions within density functional theory: *a posteriori* dispersion corrections that add energies derived from analytical expressions describing van der Waals interactions to the DFT energies and functionals containing an explicit non-local correlation contribution (so-called van der Waals functionals). Here, we will only briefly summarize the basic ideas behind these approaches, and discuss the effect they have on interface calculations. A comprehensive discussion of these approaches goes beyond the scope of this work, and the interested reader is referred to pertinent reviews.^175,176,178,189,241–248^

#### 4.2.1 *A posteriori* correction schemes

Today, the most common way to account for van der Waals interactions is *via* pairwise-additive dispersion correction schemes.^249–253^ Here, the vdW energies are computed *via* an analytical expression after the electronic self-consistency cycle has been converged. This approach is justified by the observation that, with a few exceptions,^254^ vdW interactions have a very small impact on the electron density. In their simplest form, the vdW interaction energies (*E*_vdW_) are given as1
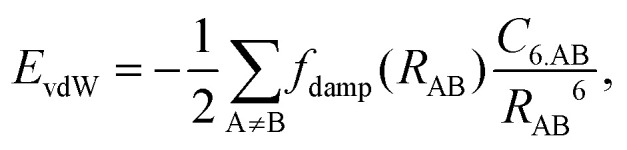
where the sum runs over all possible combinations of atoms A and B. *R*_AB_ is the distance between those atoms, *C*_6_ the effective interaction parameter and *f*_damp_ an (empirical) damping function. Several different dispersion correction schemes exist, which vary (mainly) in their approach for creating dispersion correction coefficients (*C*_6_) that reflect the chemical environment of an atom in a molecule or solid. The most commonly used schemes for hybrid inorganic–organic interfaces are that of Grimme (the DFT-Dx^250^ class of vdW correction schemes, with their most recent versions DFT-D3^255^ and DFT-D4^256^), the exchange-hole dipole moment approach of Becke and Johnson,^257^ and the methods developed by Tkatchenko and coworkers (vdW(TS)^251^ and its successors vdW^surf^^161^ and MBD-NL^258^).

The Grimme-type correction schemes were the earliest widely adopted correction schemes. In their earlier, now outdated variants (DFT-D^249^ and DFT-D2^250^), they rely on fixed coefficients for a given atomic species, neglecting the impact of the chemical environment. More contemporary variants renormalize the tabulated *C*_6_-coefficients based on the molecular geometry, which substantially boosts the accuracy of this scheme.^255^ The Tkatchenko–Scheffler-type corrections obtain the *C*_6_ coefficient from the local polarizability of the atoms, and rescale the interaction parameters based on a Hirshfeld charge partitioning scheme in order to account for the chemical environment of the atoms.^251^ For both pairwise-additive schemes, several improvements over the basic variants exist. This includes variations of the D3 scheme to account for effects beyond the dipole-approximation,^248^ three-body interaction schemes,^259^ or the use of alternative damping functions.^252^ For both the DFT-D and the vdW(TS) scheme variants exist which implicitly account for the screening of dispersion interactions in extended metals *via* modified parameters.^161,260^ Importantly, both schemes have been specifically reparametrized for interfaces, which has been done separately for metal substrates, like in the vdW^surf^ scheme,^161,261^ and for ionic crystals and surfaces.^262,263^ Using these reparametrized schemes seems highly advisable: For metals, because the corresponding parameterization partly restores the correct physics;^161^ for ionic crystals, because using the conventional parametrizations yields incorrect lattice constants or causes unphysical surface reconstructions. The vdW^surf^ scheme has shown reliable performance in describing the adsorption structure of large conjugated molecules at metal surfaces, but yields overestimated adsorption energies.^176,264^ An extensive list of parameters for the vdW^surf^ scheme has been published.^265^

When used out of the box, both the Grimme and the Tkatchenko–Scheffler approaches have known difficulties treating systems which undergo strong charge transfer, which is a common scenario at hybrid organic inorganic interfaces.^248^ In principle, D3 allows dealing with atoms that are far from neutral (*i.e.*, that experience strong charge transfer), but this requires manual tinkering with the reference systems used for the parameterization.^262^ This situation has been recently improved with the D4 variant, which rescales the interaction parameters based on an atom-in-molecule charge partitioning scheme.^266^ One could naively expect that the vdW(TS) scheme would automatically capture charge-transfer effects, since it relies on a charge-partitioning scheme. However, the Hirshfeld partitioning sometimes fails to yield correct charges (especially for negatively charged moieties and cases of strong charge transfer). The reason for this is that in the Hishfeld partitioning scheme the density is partitioned according to the densities of free, neutral atoms. Thus, the parameters in the vdW(TS) scheme are not sufficiently rescaled in cases, where a free ion would be a much better reference.^267^ Here, the situation can be substantially improved by using an iterative Hirshfeld scheme,^268,269^ which includes such an improved reference combined with an interpolation of tabulated *C*_6_ parameters between neutral and charged free-atom species. In fact, we observed for complex interfaces with multi-component adsorbate layers comprising organic acceptors and alkali metal atoms that this approach yields clearly more accurate adsorption heights^270^ (compared to X-ray standing wave experiments) than simulations based on the standard vdW^surf^ parameters.^125^

The Many Body Dispersion (MBD) method by Tkatchenko and coworkers can be seen as a method that neither fits into the pairwise additive methods nor the non-local correlation functionals.^271^ Based on the vdW(TS) or vdW^surf^*C*_6_ rescaling approach,^161^ the MBD method and its variants^272,273^ create a set of quantum harmonic oscillators at the positions of the atoms, parametrized by atomic polarizabilities derived from Hirshfeld partitioning. These oscillators are coupled within the dipole approximation to calculate a long-range correlation energy. Although this approach has shown promise for hybrid organic–inorganic interfaces,^274^ the description of the polarizability response *via* atom-localized harmonic oscillators is not able to properly capture the collective polarizability response of metals. A recent, non-local extension of the MBD correction (the MBD-NL scheme)^258^ exists that replaces the atom-in-molecules parametrizations of atomic polarizabilities with a non-local functional to compute the polarization.^275^ The approach remains to be systematically tested, but it is expected that this correction is universally applicable to molecules and solids^258^ and, thus, should perform well for hybrid inorganic–organic interfaces.

#### 4.2.2 Non-local van der Waals functionals

An alternative approach to include long-range van der Waals interactions is to use an xc functional that directly captures non-local correlation. At the same time, it should avoid a summation over unoccupied states, in order to keep the functional computationally tractable. Today, most non-local van der Waals functionals contain correlation of the form2
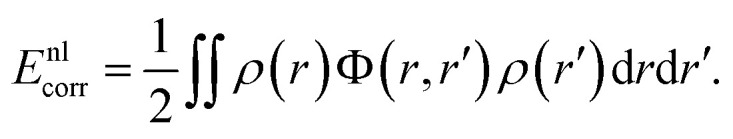
Here, *ρ* is the electron density and *Φ* is a pre-computed kernel. Different types of functionals differ in the exact form of *Φ*, which determines how the physics of non-local correlation is captured. Modern functionals base it on the local polarizability, which, for algebraic reasons, is approximated in a way that neglects screening effects.^242,248,276,277^ Like the post-processing schemes, the non-local correlation can, in principle, be added to any xc functional, although this is much less straightforward here. However, the non-local form (requiring a double integration over space, similar to exchange in hybrid functionals) made their application within periodic boundary condition calculations expensive. It was shown that a reparameterization of the local exchange and correlation energy can substantially improve the accuracies of vdW-DF.^245^ The recent reparameterizations by Vydrov and van Voorhis (VV09 and VV10) made these functionals computationally more efficient.^278,279^ In contrast to the post-processing schemes, no dedicated re-parameterizations of the kernels for interfaces exist, nor should they conceptually be necessary. A new generation of vdW-DF methods has recently been proposed, that is specifically build to deal with various competing interaction mechanisms.^280,281^ However, to the best of our knowledge, their performance has not yet been systematically assessed in the context of hybrid interfaces.

### 4.3 Consistency of the computational method and benchmarks

Many of the aforementioned functionals, combined with various dispersion-correction methods, have been applied to interfaces, including interfaces between rather large organic molecules and metallic or semiconducting substrates. Their success varies from case to case, but generally, all modern approaches for treating long-range dispersion interactions tend to perform reasonably well. However, that is not to say that all approaches yield the same results. Anecdotally, here we report the impact of the computational method on the structure of for PTCDA on Ag(111) (based on ref. 176 and 282), for which also a wealth of experimental information exists.^283^

**Fig. 10:**
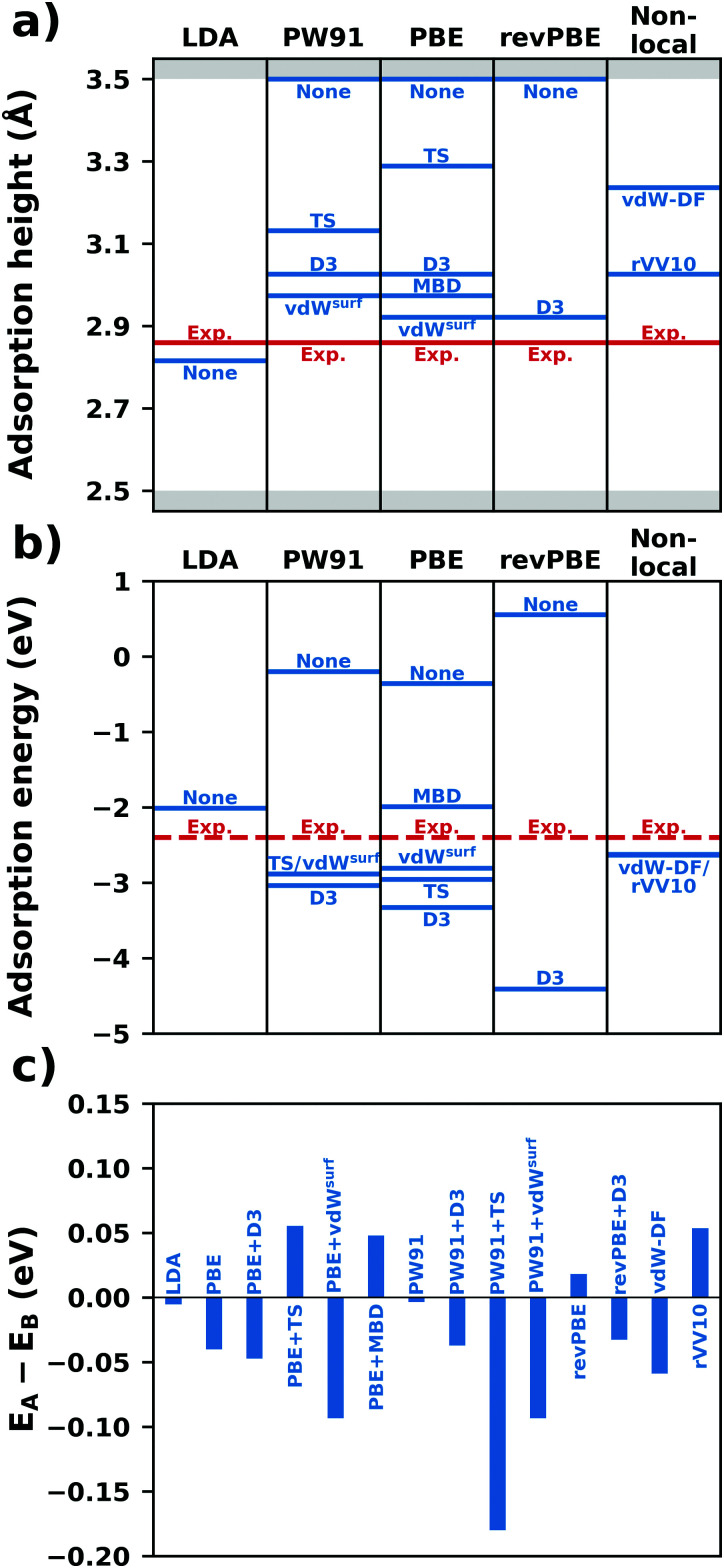
Optimized adsorption heights (a) and corresponding adsorption energies (b) for PTCDA/Ag(111) with different methods. Results for different functional/vdW-correction scheme combinations. The different functionals (LDA, PW91, PBE, revPBE), were combined with the Grimme-D3 scheme and the Tkatchenko–Scheffler correction method in their original (TS), surface (vdW^surf^) and MBD variant. The vdW-DF and the SCAN+rVV10 functional were used as representatives for non-local functionals (right column in a and b). (c) shows the energy difference between the two stable adsorption sites of the molecule. Calculations details are given in Hörmann *et al.*^282^ The experimental values are reproduced from literature:^161,284,285^ For the adsorption height, they were obtained by X-ray standing wave experiments,^284,285^ the adsorption energy was extrapolated based on the chemically related NTCDA molecule.^161^

Fig. 10a shows the adsorption height obtained with various functional/vdW-correction combinations. For the chosen system pure GGA-functionals (PW91/PBE/refPBE) yield the unphysical result that PTCDA barely binds. Conversely, combining the GGAs with the D3 or the vdW^surf^ correction yields results in rather good agreement with experiments,^284,285^ as does the non-local VV10-functional. The values for the adsorption height (*ca.* 3.05 Å) are similar to those obtained with the Random Phase Approximation.^176^ The more outdated vdW-DF and the vdW(TS) methods also yield adsorption geometries that are reasonable, but deviate more significantly from the experiment. Similar conclusions regarding the differences between the functionals are reached for the adsorption energies shown in Fig. 10b, although for the adsorption energy the “experimental value” should not be taken at face value (as it had to be extrapolated from a different molecule).^161^ Nevertheless, it becomes clear that the values obtained with the different methods scatter notably. Already within the PBE functional, the different vdW-correction schemes yield adsorption energies between −2.0 eV and −3.5 eV. The two tested non-local functionals SCAN+VV10 and vdW-DF, as well as PW91 with various correction schemes, are also within this energy window. Unfortunately, these energy differences are not just rigid offsets between different methods, but affect different adsorption positions differently. We illustrate this again for PTCDA, which has two inequivalent stable adsorption positions when adsorbed on Ag(111). They differ mostly with respect to their orientation relative to the substrate.^283^ As shown in Fig. 10c, the relative energies between the two minima assume values between −150 meV and +50 meV depending on the chosen approach. That means that even the relative ordering becomes methodology-dependent. Interestingly, Fig. 10c suggests that these variations depend more strongly on the vdW-correction than on the underlying functional.

On a more positive note, at least qualitatively the potential energy surface for interfaces seems to be relatively insensitive to the method. As shown in the Supporting Information of ref. 165, almost all methods consistently yield the two minima with very similar alignments of the molecules with respect to the substrate. Some of the methods yield additional, very shallow and energetically higher lying minima. Those geometries are not observed in the experiments, but that does not necessarily mean that they are not real.

Although PTCDA/Ag(111) is just one example, the considerations above show that the choice of the method matters. This has two important implications for practical purposes. First, it shows that the literature results obtained with different methods cannot be directly compared, at least not on a quantitative basis. Secondly, if different methods yield different results, the natural question arises whether there is one method that is clearly better than the others and could, therefore, be recommended for interface calculations?

We reckon that for a definite answer to this question not enough systematic tests have been performed to date (which is also not straightforward considering that the number of reliable experimental datasets is limited). In practice, at the time of this writing, the largest body of data exists for the PBE+vdW^surf^ method. For metallic substrates and both physisorbed and weakly chemisorbed organic adsorbates, it usually gives good results for the electronic structure^212^ (work function within 0.2 eV of the experiment) and the adsorption height (typically within 0.2 Å of the experiment).^176^ Similarly good results for interfaces have been obtained using PBE+D3.^260,286^

### 4.4 Basis sets (plane waves *versus* atom-centered)

In addition to the choice of the structural model and the computational approach (which for interfaces typically boils down to the choice of the functional and the van der Waals correction), it is also relevant to decide onto which basis functions the (Kohn–Sham) orbitals are to be mapped (which, in practice, is typically another factor that determines which computational code is applied). Most modern band-structure codes can be separated into two different classes depending on the type of basis functions they use to represent the electronic structure: One type are plane-wave-based approaches, which rely on delocalized basis functions and where the basis can be systematically improved to achieve monotonic convergence. However, they require a special treatment for the core electrons. For a detailed discussion of the various forms of that treatment, such as pseudopotentials,^287,288^ projector-augmented waves (PAWs),^289^ or linearized augmented plane waves (LAPWs),^290^ the interested reader is referred to pertinent reviews and books. The other type are atom-centered basis functions, which are centered on the nuclei and straightforwardly describe core electrons, but for which no clear systematic improvement schemes exist, as the convergence of results with basis-set complexity is typically not strictly monotonic. For sparse systems (such as surfaces exposed to vacuum) and low-dimensional systems, atom-centred basis functions have the benefit that basis functions are only placed around atoms and do not cover vacuum regions. We note that also a variety of other types of basis sets exist (and especially real-space grid representations appear to gain traction),^291–294^ but these will not be considered here. As shown by the seminal paper by Lejaeghere *et al.*^295^ for bulk materials, simulations employing plane-wave and atom-centered basis functions give the same results, provided well converged settings are used. Note that convergence is always defined with respect to a certain observable. This means that the mere fact that convergence has been reached for a given property does not mean that this applies to all quantities of interest, as discussed in more detail in Section 5. It is, thus, generally important to perform careful convergence tests, that ensure that the chosen basis set, as well as related settings, such as cutoff potentials and Brillouin zone sampling, affect the quantities of interest for a specific study by less than an “acceptable” error margin.

Thus, it is interesting to discuss briefly how the expected errors due to underconverged settings differ for the two types of basis sets, when it comes to the computational description of interfaces. Two issues deserve particular attention: (i) The description of the electron density at the surface and its decay into vacuum, which determines the interface dipole and, thus, the sample work function, and (ii) the adsorption energy of a molecule on the surface.

#### 4.4.1 Influence on the interface dipole

**Fig. 11:**
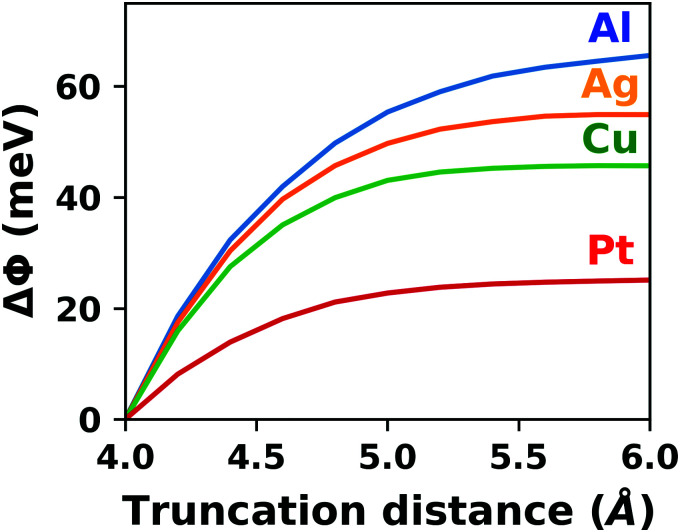
Change of the work function (Δ*Φ*), relative to the default truncation distance of 4.00 Å, for different (111) metal surfaces as a function of the employed truncation distance (increased from the default value of 4 Å) for atom-centered basis functions, calculated with FHI-aims using the PBE functional and a 33 × 33 × 1 *k*-point grid. More details and the full calculations can be found at https://dx.doi.org/10.17172/NOMAD/2020.12.07-2.

As a first step, the implications of the choice of the basis set for the interface dipole shall be discussed: By definition, plane waves are periodic basis functions that encompass the whole space. At an interface, the same basis functions are, therefore, responsible for describing the electron density in the slab and the (lack of) electronic density in the vacuum region. Because the number of used basis functions is finite, their values in the vacuum region do not perfectly cancel. This leads to patches of spurious electron density. Although that electron density is very small, under certain circumstances it can have a noticeable impact on the electrostatic potential and the work function. The spurious electron density can be reduced by increasing the number of plane waves and by a careful convergence of the SCF procedure, as will be discussed in more detail in Section 5. Conversely, atom-centered basis functions (which exist in many flavors, *e.g.*, tabulated numerically or as Gaussian-type basis sets) are only present in the vicinity of matter. Thus, with this approach, no electron density far away from the slab can exist. However, for computational efficiency, atom-centered basis functions are truncated at a certain distance from the nucleus. This reduces the number of overlapping basis functions in regions where the overlap would be very small anyways, and in this way the natural sparsity in real-space can be used to significantly speed up calculations. For surfaces, this truncation limits how far electrons can spill out from the surface. However, accurately describing this spill-out is absolutely crucial for correctly describing the surface dipole (see Fig. 11), especially for metallic substrates. It can also subtly affect the tail of the electron density above the surface, which is often used to interpret STM topographs within Tersoff–Hamann simulations.^296,297^ Particular care has to be taken here, as the default values for the truncation distance in most codes are designed for densely-packed bulk materials and can, thus, be too small for surfaces. Consequently, atom-centered codes tend to yield too small work functions and interface dipoles. As can be seen from Fig. 11, this can be solved by increasing (and converging) the truncation distance manually.

#### 4.4.2 Influence on the adsorption energy

Adsorption energies are defined as the total energy of the combined system minus the substrate and the isolated molecule(s):

3 *E*_ads_ = *E*^combined^ − *E*^substrate^ − *E*^molecules^

When calculating the energy of the combined system, atom-centered basis functions are prone to basis set superposition errors (BSSE).^292^ This well-documented effect^300^ is a consequence of incomplete basis sets and arises from the fact that in the combined system the adsorbate will “borrow” basis functions from the substrate to improve the description of its own electron density, and *vice versa*. In other words, the combined system has a larger effective basis set than the individual subsystems, which artificially lowers the energy of the combined system and leads to an overestimation of the adsorption energy. The BSSE becomes particularly relevant when using highly correlated methods. It can be mitigated by either using a sufficiently large basis, or by employing the counterpoise correction scheme (using the full basis set of the combined system also when calculating the subsystems using “ghost atoms”),^301^ In this context it should, however, be mentioned that the suitability of this method has recently been controversially discussed.^302^

**Fig. 12:**
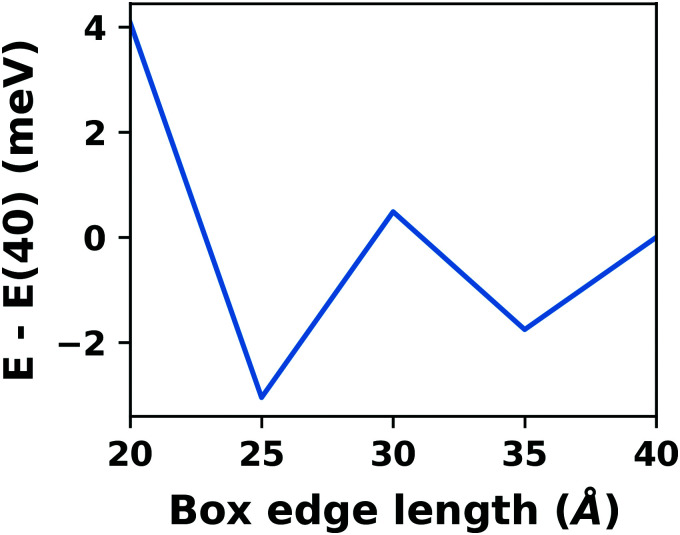
Total energy of a TCNE molecule placed into a cubic box of increasing size, relative to the value obtained for the largest box (with an edge-length of 40 Å). The results were calculated with VASP^298,299^ using the PBE functional and the default cutoff energy of ∼290 eV for the plane waves. More details and the full calculations can be found at https://dx.doi.org/10.17172/NOMAD/2020.12.07-4.

When employing plane-wave basis sets, the number of plane wave basis functions depends on the chosen cutoff value as well as on the size of the unit cell. The number of atoms, *per se*, does not play a role. Therefore, calculations with such basis sets are principally BSSE-free. For plane-wave basis sets, the challenge when calculating adsorption energies occurs for the isolated molecule as reference system. Since (with few exceptions) plane wave calculations require periodic boundary conditions, in practice, the isolated molecule is calculated as a single molecule in a large unit cell, electrostatically decoupled by dipole- and quadrupole corrections schemes in all spatial directions. However, since the cell needed to decouple periodic replicas might be different from that of a tightly packed combined system, the basis set used for the description of the isolated molecule is usually not the same as the basis set for the combined system and for the substrate. The impact of varying the size of the unit cell for a given cutoff energy is exemplarily illustrated for the case of the TCNE molecule in Fig. 12. The energy variation is rather small (amounting to a few meV), but in the same spirit as the BSSE, this inconsistency can lead to small errors for the adsorption energy. Conceptually, this problem can be mitigated by using an overconverged basis set, *i.e.* by increasing the basis set cutoff until the energy no longer depends on the unit cell size. However, that same cutoff must be used also for the combined system, making this strategy quite costly (and, given the small error, often not worth the effort).

## 5 Algorithms, parameters and convergence: best practices for interface simulations

Once the major choices regarding the structural model, the employed xc-functional, and the basis set have been made, the simulation can be run. To solve the various parts of the Kohn-equations numerically, several different algorithms are used. A salient difference to the issues discussed in Sections 3 and 4 is that these algorithms and their settings are often somewhat hidden from the user, inasmuch as often default choices exist that are not mandatory to be specified in the input file.

As we have frequently mentioned in this work, the default choices are often sub-optimal for interfaces. In this section, we will, therefore, discuss some of the most common algorithms together with the usually applied settings. We will specifically focus on their major pitfalls when modelling hybrid inorganic–organic interfaces with band-structure based DFT, highlighting unfortunate instances where defaults can even lead to physically incorrect results. Although we use periodic boundary conditions for the examples in this section, we emphasize that the challenges demonstrated here can be analogously encountered in cluster calculations.

This section will be organized as follows: First, we will discuss the most critical settings within the self-consistent field (SCF) approach. These are (i) how to choose appropriate convergence settings, (ii) the importance of choosing the right initial guess for the electronic structure, (iii) different options to update the electronic structure (and the ideal parameters to achieve convergence fast), and (iv) the impact of level broadening and its dependence on the number of atoms in the system. We then proceed to discuss geometry optimizations, showing why sometimes completely different geometries are obtained, when using the same starting geometries, but different optimization strategies. As a final note, we demonstrate what can happen when a higher-level xc-functional is applied on top of a geometry obtained with a lower-level xc-functional.

### 5.1 SCF convergence

Within the self-consistent field (SCF) technique, the Kohn–Sham equations are solved iteratively until the results (*i.e.*, one or several properties of the system) no longer change between two subsequent iterations. When this is the case, *i.e.*, when the selected property no longer changes within a pre-defined threshold, the SCF is “converged”. In principle, any property that can be computed can also be selected as convergence criterion, including in particular the total energy, the change of the electron density, the sum of eigenvalues, *etc.* In practice, many codes only consider the total energy (and, for geometry optimizations, the forces on the atoms). For details on the inner workings of SCF algorithms and the assessment of performance, we refer to the recent review by Woods *et al.*^303^

#### 5.1.1 Spurious convergence

**Fig. 13:**
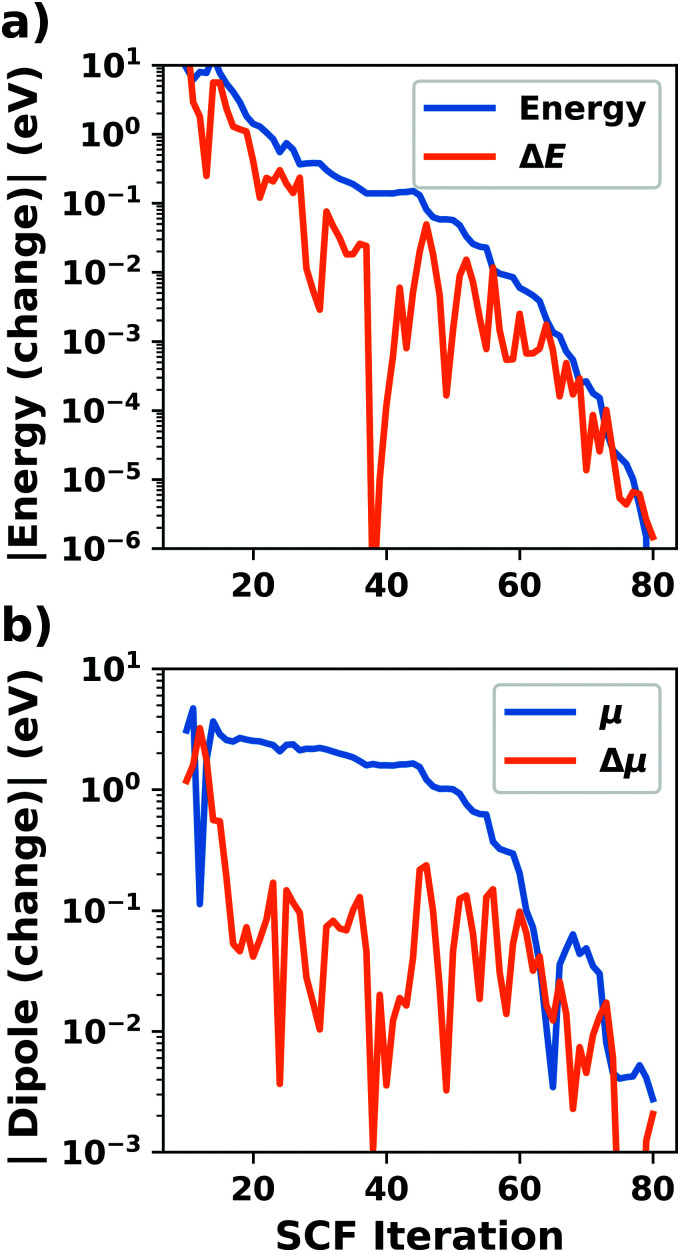
Convergence behavior of phenyl-piperazinedithiocarbamate on Au(111) using reasonable but non-optimized settings. (a) Evolution of the energy (relative to the last energy given by the calculation) and the (absolute of) the change of the energy between subsequent iterations. (b) Evolution of the dipole moment (again relative to the last value given) and the change of the dipole moment between subsequent iterations. The original calculation and its settings can be found at https://dx.doi.org/10.17172/NOMAD/2020.12.07-5.

To illustrate two potential pitfalls, in Fig. 13 we show the evolution of the total energy and the dipole moment for a system we recently investigated, a phenyl-piperazine based self-assembled monolayer bonded to the Au surface *via* a dithiocarbamate anchoring group.^304^ For this example, the SCF was run with parameters that were “inherited” from a previous calculation (*i.e.*, performed well there). The details of the settings are not relevant here (they can be found at https://dx.doi.org/10.17172/NOMAD/2020.12.07-5), except that the SCF was set to converge when the energy change between subsequent iterations fell below 10^−6^ eV (a common, tight default) and to run for at least 60 steps.

Most importantly, we note that had we used only the change in energy as a threshold (orange line) as criterion, the calculation would have stopped at iteration 38. At this point, the SCF visited, by coincidence, two solutions to the Kohn–Sham equations that are almost identical in energy, leading to an apparent, spurious convergence. However, as can be seen from the blue line, the absolute energy (plotted relative to the energy at the last iteration) is still significantly above that of the fully converged energy (obtained at iteration 79 here). Such situations are rare, but not very. It is, therefore, generally advisable to inspect the SCF evaluation after each calculation. Sudden, surprising drops of the energy change are often an indication for spurious convergence and should be critically second guessed. In order to avoid this behavior in the first place, it is useful to employ additional convergence criteria (if the code allows), such as criteria for the change of the electron density or the sum of eigenvalues, as it becomes increasingly unlikely that multiple of these thresholds are met simultaneously unless the solution has approached the true minimum.

As second point, Fig. 13b illustrates that the convergence of the interface dipole does not directly correlate with the convergence of the energy. If our energy threshold for the energy change had been 10^−4^ eV (which is often sufficient to obtain reasonable energies), the calculation would have finished at SCF step 69 (the spurious convergence at iteration 38 notwithstanding). However, at this point the interface dipole would still be approximately 50 meV away from the value obtained when converging the calculation to an energy threshold of 10^−6^ eV (see blue curve). Even when continuing the SCF until the threshold of 10^−6^ eV is reached, the dipole still changes several meVs between the last iterations. This indicates that in this example, even using the relatively tight convergence criterion above may have been insufficient to obtain a fully converged value.

#### 5.1.2 Convergence thresholds

We can now ask, which convergence threshold for the total energy would be ideal to obtain a numerically accurate result with as few iterations as possible. For this, we must first discuss how accurate our result needs to be. When comparing theory and experiment, it is rarely necessary to obtain energies with an accuracy better than 1 meV, so this is chosen as target accuracy. In fact, as we discuss below, for interfaces the limits imposed by the various numerical approximations (sampling of *k*-space, density of the integration grid, basis set, *etc.*) usually limits the numerical accuracy for the total energy to something on the order of 0.1–1 meV per atom, which translates to roughly 1–100 meV per unit cell, making a much tighter target accuracy for energies obsolete. Once could, therefore, ask if this makes significantly higher convergence criteria obsolete.

**Fig. 14:**
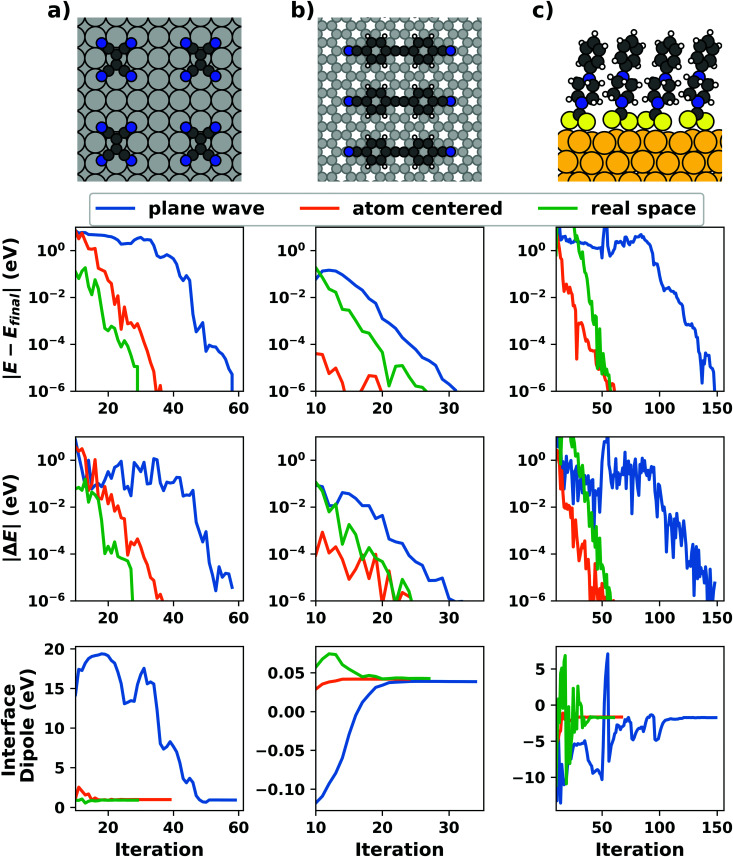
Convergence behavior of the SCF for three model system using the plane-wave codes VASP, GPAW version 19.8.1b1 with a real-space grid, and the LCAO code FHI-aims. The different systems are (a) TCNE on Ag(100),^163^ (b) phenyl-piperazinedithiocarbamate on Au(111)^304^ and (c) bis-cyanophenyl on graphene.^305^ From top to bottom, the panels show an atomistic representation of the simulated system, the evolution of the final energy (relative to the energy obtained after converging the SCF to a threshold of Δ*E* < 10^−6^ eV), the change of the total energy between two subsequent iterations, and the dipole moment per unit cell. For all numeric settings, the default values as supplied by the codes were used. We emphasize that the results should not be mistaken as benchmarks, because (a) the time required for the iterations differs between the different packages, and (b) because when choosing smarter algorithms and settings within each code, the number of iterations could be substantially optimized.

To suggest guidelines for choosing an appropriate threshold, we exemplarily look at three different systems we have investigated in the past: (a) A tetracyanoethylene (TCNE) molecule lying flat on an Ag(100) slab,^163^ (b) a phenyl-piperazine based self-assembled monolayer bonded to the Au surface *via* a dithiocarbamate anchoring group,^304^ and (c) a graphene sheet with an adsorbed 4,4′-bis(phenylcarbonitrile) molecule.^305^ The three systems are shown in the top panels of Fig. 14a–c. To illustrate how diverse the SCF procedure evolves, we re-calculated all of these systems using a plane-wave basis set,^298^ an atom-centered basis,^306^ and a regular real-space grid (using three different codes).^307^ For the sake of illustration, we strictly adhered to default settings, rather than optimizing the input file (except for switching on the dipole correction and enforcing an SCF threshold of 10^−6^ eV for the energy). Therefore, we emphasize that the results should not be mistaken as benchmarks, because (a) the time required per iteration differs between the different packages, and (b) because when choosing smarter algorithms and settings within each code, the number of iterations could be substantially optimized. Instead, the point of the comparisons in Fig. 14 is to show how different the convergence of different approaches can be.

When comparing the energy change between iterations Δ*E* (third row) with the value of the total energy (second row), it becomes clear that a given accuracy for *E* is typically obtained when Δ*E* is approximately one order of magnitude smaller (*e.g.*, to obtain an energy accurate to within 1 meV, Δ*E* should be 10^−4^ eV or less). Notably, that statement only holds when the SCF has reached a state of “exponential convergence”, where most iterations yield ever smaller values of Δ*E* (as opposed to “sideways movement” for the energy, as observed, *e.g*., for the blue line in the first 100 steps for system c). As above, we recommend to manually inspect the SCF behavior to assert that this exponential convergence region has been reached.

From the fourth row of Fig. 14, which shows the dipole moment, it becomes clear that the three approaches visit very different regions of the electronic potential energy surface before finding the minimum energy solution. In particular, the plane wave basis (blue line), has relatively large values for the dipole in the first steps. This behavior is a consequence of the basis set type (with a finite cutoff): The degree to which the total energy is converged is primarily determined by the electron density in the slab. As discussed in Section 4.3, in plane-wave calculations there are, however, also some small but non-zero amplitudes for the total wave function in the vacuum region. This spuriously places electron density into vacuum and, due to the overall charge neutrality of the unit cell, abstracts electron density from the slab. As the energy contribution of this dipole is very small (as discussed in Section 3), the SCF experiences only a weak “driving force” to remove this spurious dipole. We emphasize that this (only apparently inefficient) convergence behavior can be substantially improved by playing with the basis set cutoff and the size of the vacuum.

At the same time, the fourth row of Fig. 14 demonstrates that obtaining a converged energy does not necessarily result in robust and converged values for other quantities, *e.g.* a Δ*E* threshold of 10^−4^ eV does not mean that the interface dipole is converged to 1 meV. Because, in fact, most electronic properties do not directly correlate with the total energy, it is generally advisable to monitor not only this quantity, but also the quantity of interest during the SCF procedure. In fact, the best practice would be to include an explicit convergence criterion for the primary quantity of interest directly into the SCF cycle. *I.e.*, when calculating adsorbate-induced changes of the sample work function, requiring the unit cell dipole to converge as one of the criteria to exit the SCF cycle is a very useful strategy. Although this can generally be implemented with only a few lines of code, unfortunately it seems that the convergence of the unit cell dipole is only rarely monitored or used as an SCF convergence criterion in practice.

In passing, we note that in the seminal work by Lejaeghere *et al.*, it has been established that all three codes used here give consistent results for energy-derived values^295^ when using properly converged settings. Still, using default settings (and a Δ*E* threshold of 10^−6^ eV, as commonly done in literature), we find that the interface dipoles vary by approximately 5–10% between the three approaches/codes for the three studied systems. This certainly has to be kept in mind, when comparing results found in literature and when comparing computed to experimentally obtained values.

### 5.2 Initial guess for the SCF procedure

The initial guess for the SCF cycle provides the starting point for any calculation. Trivially, the closer the starting point is to the correct electron density, the fewer iterations will be required to complete the calculation. However, the starting point can also have a qualitative impact on the outcome of the calculation: In cases where multiple minima for the electronic structure exist, the initial guess for the SCF decides into which solution the SCF will converge. We found this to be of practical relevance for magnetic and open-shell systems. One anecdotal example is the adsorption of the (open-shell) molecule Cu-phtalocyanine on Ag(111).^308^ There, when initializing the calculation with a spin of 1 μB or larger on the central ligand, it converges into a metal-centered state. Conversely, when non-zero initial spins are set on both the central atom and the four closest nitrogen atoms, the result is a more extended charge transfer, involving the ligand as well as the central atom. The two different states also show a different overall multiplicity: While the metal-centered state is a doublet, the state involving charge transfer to the ligand has triplet multiplicity.^308^

Typically, the initialization of the SCF procedure relies on one of two strategies: The method traditionally used by most quantum-chemistry codes, which are geared towards molecules and employ open boundary conditions, is to provide a guess of the wave function obtained by an approximate method that does not need to be calculated iteratively. Examples would be a guess derived from the extended Hückel method,^309^ the Harris functional,^310^ or the diagonalization of the core Hamiltonian. From the guess wave function, the electron density (and/or the density matrix) is constructed. The major advantage of this strategy is that it provides a high level of control over the initial guess, as the population of each orbital can be manually specified. The downside is that such methods are heavily parameterized and that suitable parameters often do not exist for heavy atoms or non-trivial bond topologies, as encountered at interfaces. Therefore, wave function-based guesses are rarely used in band-structure codes.

Instead, most band structure codes rely on an initial guess for the electron density, rather than the wave function, for which the (initial) basis set coefficients are initialized at random. The electron density guess is commonly obtained using the so-called superposition of atomic densities (SAD).^311,312^ Here, for each atom in the system a hypothetical, non-interacting electron density is calculated by solving the corresponding radially-symmetric Kohn–Sham equations. The first guess of the electron density is then given by the sum of all individual electron densities. This method has the advantage that it provides a relatively good idea about the electron distribution of the core electrons and even a rough description of the valence electrons. However, it does not contain chemical insight, *i.e.* the initial guess is agnostic of the bonding environment. Consequently, for metals, where the bonding is (mostly) isotropic, SAD provides a reasonable first guess for the electron density, but it performs less well for covalently bonded systems, such as molecules.

We note in passing that a similar concept is applied when using the Harris functional, where the energy is calculated non-self consistently from an input density that is constructed as the sum of the densities of the system constituents.^310^ However, the choice of constituents is *a priori* arbitrary, and should be done such that they only interact weakly with each other.^310^ For molecular crystals, it has been shown to be prudent to use the pre-converged electron density of individual molecules (rather than atoms), to obtain good results.^313^

The SAD method has two challenges that should be kept in mind. First, by construction, the dipole moment of a SAD guess is zero, as for all atoms the charges in the nuclei are exactly compensated by the charges in the spherically symmetric electron shells. Especially for interfaces with substantial charge transfer the electrostatic potential of the initial guess can, therefore, differ significantly from the potential of the final solution. Consequently, because the electrostatic potential affects the relative ordering of the orbitals (and their occupation), the initial guess is often far away from the converged situation. This can lead to a slow convergence or even a divergence of the SCF procedure. In practice, this is usually indicated by strong oscillations of the electron density and dipole moment between subsequent iterations. As we show below, this can often be mitigated *via* a strong damping of the SCF employing small mixing coefficients.

The second challenge arises for open-shell systems. Usually, by default the SAD density will be of singlet multiplicity with an equal amount of spin-up and spin-down electrons. *A priori*, this will also be the case for systems in which the real situation is different, like for magnetic systems or (weakly interacting) singly charged molecules, *i.e.* cations or anions, at interfaces. The main problem arising from that initialization is that for symmetry reason, singlets are always stationary points on the electronic potential energy surface (either true minima or saddle points).^314^ Hence, different spin solution can only be obtained by preparing an initial guess with a net spin moment. Unfortunately, the SAD approach allows only limited control here. Since orbitals/bands cannot be directly addressed, the initial guess must be constructed by assigning an “initial” spin moment to each atom, which is not necessarily straightforward, even if the spin multiplicity of the unit cell is known. Thus, even when the initial guess has the correct multiplicity, it can be far from the correct solution. In fact, since semilocal functionals only have relatively small barriers between stable spin solutions (due to the lack of static correlation, as shown by Cohen *et al.*^314,315^), it is easily possible to converge to a solution with a total spin moment different from the desired one.

### 5.3 Density/wave function update algorithms

Once the initial wavefunction (and/or electron density) has been set up, the SCF algorithm solves the Kohn–Sham equations for the given charge distribution yielding a new wavefunction (and/or electron density). It turns out that if one uses the output directly as input for the next iteration, convergence is hard to reach, as SCF iterations can show highly oscillatory behavior or very slow convergence. Typically, the step size between consecutive SCF steps is restricted by only allowing the density to deviate by a certain amount from the previous step. To achieve faster convergence of the SCF, state-of-the-art programs sacrifice guaranteed convergence for performance using various acceleration algorithms. In this work, we will consider three of those, that are often used together: The density mixing method (with the specific example of the Pulay method),^316^ Charge Preconditioning, and Level Broadening. All of these algorithms have the potential to yield efficient convergence, but require parameters that ought to be (somewhat) adapted for each system. Unfortunately, the optimal parameters differ significantly for metals, molecules, and interfaces, as will be described below.

#### 5.3.1 Density mixing

The simplest of these strategies is linear mixing (also known as naïve mixing or underrelaxation), where a linear interpolation between the input guess and the output wavefunction/density is taken as new input:

4 *n*_*i*+1_ = *n*_*i*_ + *α*Δ*n*

Here *n*_*i*_ is the electron density of the *i*th iteration (*i* + 1 being the next iteration), Δ*n* the difference between the input and the output of the present SCF step, and *α* the mixing parameter. This strategy bears the important advantage of guaranteed convergence, if very small mixing coefficients are employed. However, since small mixing parameters require a very large number of iterations, it is too costly to rely exclusively on this approach for interfaces. Still, it can be useful for systems that are hard to converge. If the initial guess is particularly bad (*e.g.*, when using SAD-densities for systems with very large interface dipoles), it is useful to employ linear mixing for the first few (5–10) iterations. This allows the SCF to reach a “harmonic” part of the solution space, where the more sophisticated approaches described below can take over.

To accelerate convergence, many codes replace linear mixing with various forms of nonlinear density mixing. Different approaches include the Broyden^317^ and the Pulay scheme, which is also known as direct inversion in iterative subspace (DIIS) and which is the most prevalent convergence acceleration scheme.^316,318^ While a full mathematical explanation of the DIIS scheme is beyond the scope of this contribution, the core idea is readily explained. In every iteration, the “residual”, *i.e.* the deviation of the charge density from the (expected) converged situation, is computed and stored. After a given number of iterations, the residuals from the previous iterations are combined in a coupled set of equations to determine the point on the electronic potential energy surface for which a zero “length” of the residual (the “null vector”) is expected. This works best if the electronic structure is already close to the converged results, *i.e.* in the “harmonic” part of the energy surface. Conversely, when far away from the minimum, the error vectors may point to the wrong direction or change direction rapidly between consecutive steps making this approach potentially unstable.

Like in linear mixing, also in the Pulay scheme scaling is used to interpolate between the present wave function (or density) and the predicted optimal one. Pulay mixing, thus, contains two parameters, namely the number of iterations for which the residuals are stored, and the mixing parameter, in analogy to eqn (4). These define how many iterations should be stored and how much the difference between the present and the “expectedly correct” electron density should be damped.

As will be discussed in more detail below, for metals, where the SAD electron density is qualitatively close to the final electron density and the charge rearrangements within the SCF are mostly short-ranged, it is good to apply a small mixing parameter (<0.2). For molecules, where the initial guess is worse, faster convergence is usually achieved by applying larger mixing parameters (>0.4). Simulations of hybrid metal–organic interfaces, thus, face the challenge that the optimal values for the constituents differ significantly. This is amplified by the fact that many of these interfaces exhibit long-ranged charge-transfer between the substrate and the adsorbate. As this long-ranged charge transfer and its associated interface dipole shift the relative levels of the constituents, overshooting the charge-transfer in one iteration (which is then overcorrected in the next iteration) poses one of the major caveats for the SCF. Although this overshooting can be reduced using a charge-preconditioner (see below), in practice it is best to choose a relatively small mixing parameter (∼0.2, close to metal value). Generally, this will make the calculations slow (*i.e.*, require many iterations), but decrease the likelihood that the SCF exhibits strong oscillations that prevent it from converging at all.

#### 5.3.2 Charge preconditioning

Bulk metals are isotropically bonded with a highly homogenous electron density distribution. At the surface, however, the homogeneity is broken as electron density “spills out” into the vacuum, forming a surface dipole. In terms of SCF convergence for a metal slab, this means that electron density needs to be redistributed from the center of the slab to its surfaces. Despite the small values for charge mixing advised in the previous section, most charge mixing parameters tend to overshoot this long-ranged bulk-to-surface charge transfer in some SCF iteration, bringing too much electron density to the surface. In the next iteration, this is then (over)compensated, redistributing the electron density back into the bulk or possibly even to the opposite surface of the slab. The resulting oscillatory behavior of the SCF slows down or outright prevents convergence. This phenomenon is a variation of what is commonly known as “charge-sloshing”.^319^

**Fig. 15:**
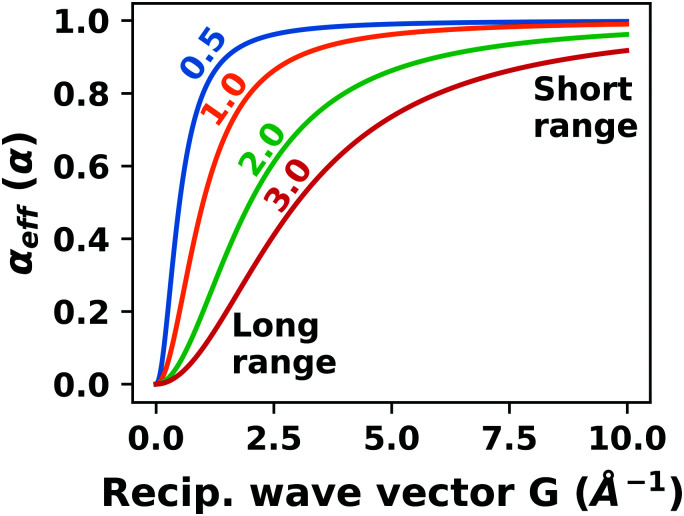
Effective mixing parameter *α*_eff_ as a function of the reciprocal wave vector *G* for different values of *q*_0_ when applying the Kerker preconditioner.

The problem of charge sloshing is routinely countered using so-called preconditioner algorithms. Multiple variants exist (*e.g.*, Kerker,^320^ Broyden,^321^ or Teter^322^ preconditioners), that all rely on the same basic idea: The charge density difference (between the SCF input and output) is transformed into reciprocal space (*i.e.*, Fourier-transformed), and the low-frequency components, which correspond to long range charge density differences, are then damped or removed. This is most easily demonstrated on the Kerker preconditioner, which has the form5
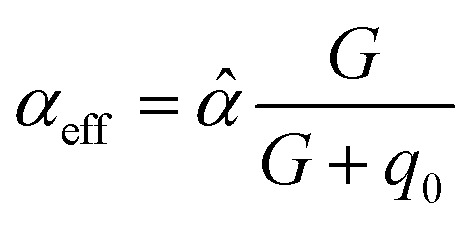
Here, the effective mixing coefficient *α*_eff_ is given by *α*, the “global” mixing coefficient of the density mixing algorithm, *G*, the Fourier component (*i.e.*, wavelength of the charge-density difference in reciprocal space), and a parameter *q*_0_. The impact of this parameter is illustrated in Fig. 15. Small values for *q*_0_ result in a relatively unperturbed mixing parameter for short-distance charge rearrangements, and a rapid decay of the effective mixing for long-range rearrangements. In other words, small values of *q*_0_ efficiently suppress long-ranged charge-transfer compared to short-range changes. Larger values of *q*_0_ lead to a smoother transition, *i.e.* they still emphasize short-ranged rearrangements over long-ranged changes, but not as pronounced. Note that at the same time, even in the short range the effective mixing parameter is reduced compared to its global value. Therefore, when choosing larger *q*_0_, it is often effective to concomitantly increase *α* in order to retain an efficient convergence behavior.

Choosing or deriving an optimal value for *q*_0_ is often challenging. For bulk materials, optimal values for the different preconditioner schemes can be deduced based on theoretical considerations. The situation is, however, more complicated when dealing with interfaces. The above-mentioned charge-sloshing results in charge rearrangements perpendicular to the surface (*i.e.*, in *z*-direction), while parallel to the surface (*i.e.*, in *x*- and *y*-direction), the slab behaves more like a bulk material. Clearly, the ideal situation would be to impose a different damping behavior in each direction. Unfortunately, most (if not all) implementations of preconditioners are isotropic. Moreover, the situation is further complicated by the fact that the magnitude of the bulk-to-surface charge transfer depends on the thickness of the slab. Depending on the implementation, even the size of the vacuum may play a numerical role. Finally, the ideal settings for the preconditioner are strongly, but non-trivially related to that of the Pulay mixer.

**Fig. 16:**
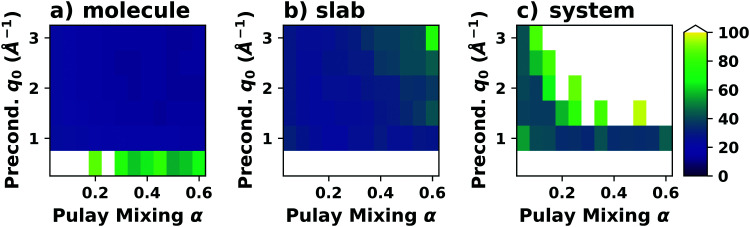
Number of iterations required for (a) the TCNE molecule (nonperiodic) alone (b) the Ag(100) slab alone, and (c) the TCNE molecule on Ag(100) to converge to 10^−4^ eV using different settings for the Pulay mixer, *α*, and the Kerker preconditioner, *q*_0_. White fields indicate that convergence was not reached within 100 iterations The calculations were done with FHI-aims using the PBE functional and a 3 × 3 × 1 *Γ*-centered *k*-grid. Further details can be found at https://dx.doi.org/10.17172/NOMAD/2020.12.07-6.

To demonstrate this issue – and to provide some general guidelines on how to choose parameters – we have tested the required iterations to converge the TCNE/Ag(100) interface (from the previous example, see Fig. 14) to Δ*E* = 10^−4^ eV using various combinations for the SCF settings (varying the Pulay scaling parameter between 0.05 and 0.6 and the settings for *q*_0_ between 0.5 and 3.0 Å^−1^). The results are shown in Fig. 16 for both the combined system and the isolated subsystems. As Fig. 16a shows, the molecule converges quite efficiently irrespective of the settings, but a large mixing parameter and a large value for the preconditioner are optimal. The Ag(100) metal slab, shown in Fig. 16b, is more sensitive to the chosen parameters (and requires more iterations) and shows the opposite behavior: Here, small values for mixer and preconditioner are beneficial.

The joint TCNE/Ag(100) system is electronically the most complicated, and, as shown in Fig. 16c, also the one most sensitive to the chosen parameters. Indeed, for this system (which is rather prototypical for interfaces), we find that not all parameter combinations allow the system to converge at all (or at least within 100 iterations). Especially the combination of a large *q*_0_ value for the Kerker preconditioner and large values for the Pulay mixing is very inefficient. Overall, we find that it follows the trends of the slab, *i.e.* mixer and preconditioner should have small values. Interestingly, combinations where either the preconditioner or the mixer have small values converge fast, and if either is small, the value of the other tends to matter less. However, (as also for the slab and the molecule), too small values for *q*_0_ (here 0.5 Å^−1^) prevent the system from converging at all, at least within the first 100 SCF steps.

In passing, we note that the energy obtained in all converged runs is highly reproducible. For the more than 200 different algorithmic settings for which the SCF converged, employing a convergence criterion of Δ*E* = 10^−4^ eV lead to a standard deviation in the final energies of only 2 × 10^−5^ eV.

#### 5.3.3 Level broadening and occupation

Another class of commonly applied algorithms to facilitate convergence are so called “level broadening” or smearing schemes. Those pursue a two-fold purpose: All (periodic) observables need to be calculated by integrating over the 1st Brillouin zone. This can be either done using broadening methods (*i.e.*, replacing the step-function for the occupation with a different function) or by interpolation, *e.g.*, using the Tetrahedron method.^323^ On the one hand, artificially broadening the energy levels allows for interpolations in *k*-space, thus reducing the number of *k*-points that need to be calculated to obtain the correct electronic structure (and, hence, the computational effort). On the other hand, for metallic systems, broadening schemes also reduce the so-called “level-switching” problem, which adversely affects the SCF convergence.

This problem occurs because at zero temperature, the Fermi function is reduced to a step function with the step at the Fermi-energy; *i.e.* all states below the Fermi-energy are fully occupied, while all states above are empty. For metallic systems, where the Fermi-energy cuts through a band, small changes of the electron density in one iteration can easily change the potential such that some eigenstates are shifted below the Fermi-energy, while others are shifted above it. In the next iteration, the electron density is adjusted accordingly, which again leads to shifts of some eigenstates from above to below the Fermi energy and *vice versa*. This can lead to notable charge rearrangements throughout the SCF, which impede convergence. The core concept of level broadening (or “smearing”) schemes is to replace the zero-Kelvin Fermi-function with a different occupation function that exhibits a smoother transition between completely empty and fully occupied states and allows for fractionally occupied orbitals. All these replacement functions contain a free parameter *σ*, that governs how fast the occupation drops from full to empty around the Fermi energy.

In passing, we note that there are two different ways to look at these schemes: either, each state is at a discrete energy and (partially) occupied according to the replacement function (*e.g.*, a Fermi–Dirac-distribution function, see below), or each state is broadened in energy (using the derivative of the replacement function) and occupied using a step function. Both viewpoints are mathematically equivalent. For convenience, most codes work internally with the former, but plot the density of states using the latter approach.

**Fig. 17:**
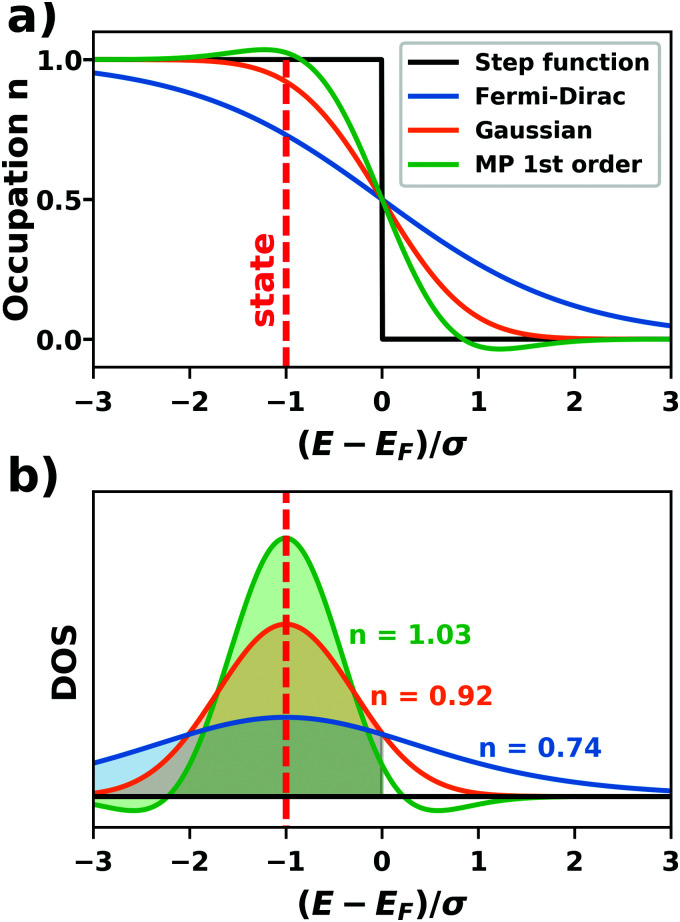
Relationship between employing level broadening and using occupation functions. Panel (a) shows the occupation number for a state at an energy 1*σ* below the Fermi energy (red dashed line) when using different occupation functions. Panel b shows the same state broadened by a corresponding smearing function (using a broadening parameter of 1*σ*). The integral over the state up to the Fermi energy is the occupation, which is indicated in the plot. Note that the smearing function is the derivative of the occupation function.

The easiest – and most straightforward – way would be to employ the Fermi–Dirac occupation function with a finite temperature. Although this does solve the “level switching problem”, it shows very unfavorable behavior for the interpolation within reciprocal space, *i.e.* it does not efficiently reduce the required number of *k*-points. Therefore, Fermi–Dirac distributions are usually replaced with a different functional form. Fig. 17 compares the shapes of some of these occupation functions for a given value of *σ*. For a given state it also illustrates the corresponding level broadenings, which yield identical occupations when combined with a step function. The most commonly used occupation functions are Methfessel-Paxton^324^ functions, which have the following general form:6
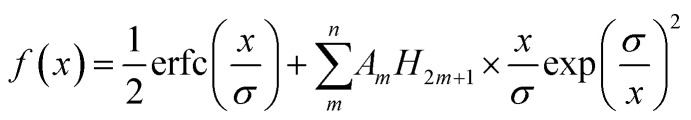
Here, the first term is the error function, *i.e.* the integral over a Gaussian function, while the second term is a sum over Hermite polynomials up to *n*’th order. 1st-order Methfessel–Paxton functions are known to show favorable convergence (with respect to the number of *k*-points) for metals. However, because they allow for negative occupation numbers, they can easily lead to unphysical results for semiconductors and particularly for molecules. For interfaces that are not necessarily fully metallic, it is, thus, advisable to use a Gaussian smearing (which corresponds to a zeroth-order Methfessel–Paxton occupation function).

**Fig. 18:**
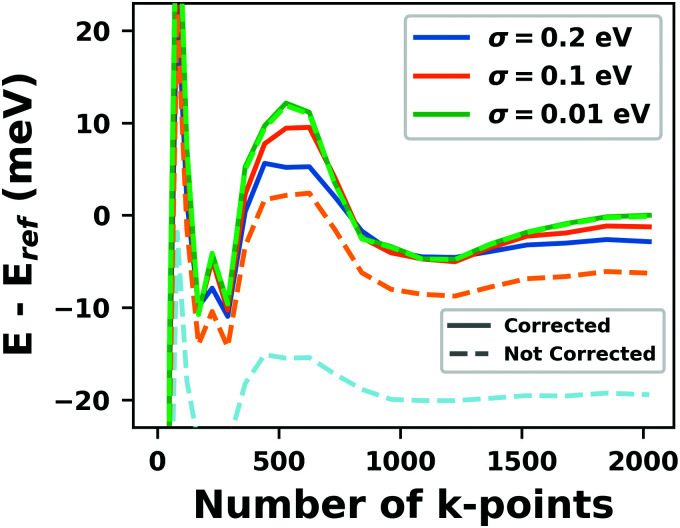
Convergence of the free energy (dashed lines, labeled as “not corrected”) and the energy extrapolated to *σ* = 0 (solid lines, “corrected”) as a function of the number of *k*-points for a 5 layer Cu(111) slab using an evenly spaced *Γ*-centered *k*-point grid and obtained using the Gaussian broadening scheme with different smearing parameters. Note that the dashed green line coincides with the full green line. All data were calculated with VASP using the PBE functional and the default value for the cutoff of 294.4 eV. Further computational details and the full results can be found in the NOMAD database https://dx.doi.org/10.17172/NOMAD/2020.12.07-7.

As mentioned above, the main advantage of using smearing methods is that the energy in the SCF procedure converges significantly faster with the number of *k*-points, *i.e.* it is possible to perform the same calculation with fewer *k*-points, and, thus, less computational effort. However, the disadvantage is that the total energy in the SCF no longer represents the “correct” total energy at *T* = 0 K (nor at any other temperature); even for a Fermi–Dirac type occupation, it would only be an ensemble-average approximation. Rather, the SCF now calculates an effective “free energy”. As shown for the example of a 5-layer Cu(111) slab (primitive unit cell, calculated using VASP) in Fig. 18, the free energy depends on the chosen value of *σ*, even for a converged *k*-point grid. Moreover, the free energy is no longer variational (*i.e.*, it can also increase during the SCF procedure). Fortunately, once the SCF procedure is converged, it is still possible to obtain the correct electronic energy by extrapolating to *σ* = 0.^299,325,326^ The corresponding energy is also the one that should be taken for all postprocessing. As shown in Fig. 18, the result after extrapolation as expected still varies significantly with the *k*-point grid but has become rather independent of *σ*.

Still, one needs to be careful when performing geometry optimizations. During the SCF, the forces acting on the atoms, *i.e.* the derivative of the energy with respect to nuclear displacements, are obtained from the free-energy, not the back-extrapolated zero-K energy. This may result in the geometry optimization requiring more steps (*i.e.*, being slow), or even yielding incorrect results. For a given calculation, the best *σ* is, thus, a balance of being large enough to require as few *k* points as possible, while not introducing too large errors in the total energy. Generally, values between 0.01 and 0.2 eV are known to produce (relatively) sensible results for the energy and the geometry.

To benefit maximally from the smearing interpolation while avoiding artificial results, the most prudent approach is to perform initial calculations (including geometry optimizations) with a relatively large broadening and few *k*-points – although not too few, as this may lead to serious artefacts, as we show below. The final results should then be verified using a smaller broadening (0.01 eV), with an appropriately higher *k*-point density.

### 5.4 Local geometry optimization

In this section, we will focus on strategies for efficiently finding the structure of the (local) energetic minimum. Local in this context means that we will only consider “conventional” geometry optimization schemes that find the minimum structure in a specific search basin of the potential energy surface which is determined by the initial guess structure. For a discussion of global structure search algorithms the readers are referred to the recent book by Oganov.^327^ At first, we will look at sensible thresholds for the geometry optimization for a simplified potential energy surface. We will then proceed to a more complex system. In particular, we will demonstrate why the choice of the initial guess for the Hesse matrix is imperative for the performance (and even the qualitative outcome) of a geometry optimization and look at error bars for adsorption heights. Finally, we will discuss strategies to make this typically rather time-consuming task as efficient as possible.

**Fig. 19:**
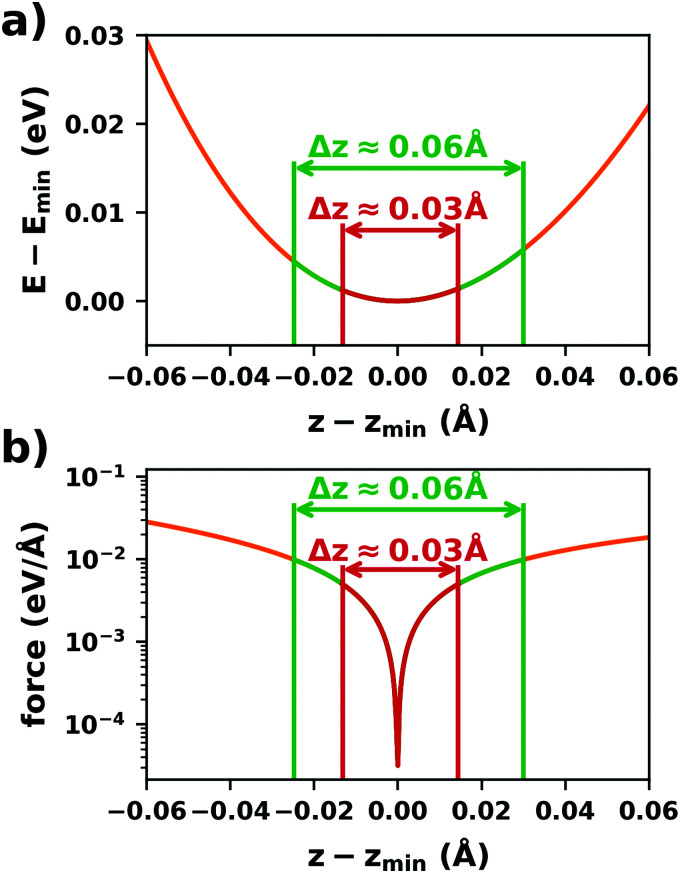
(a) Total energy (relative to the minimum) of PTCDA on Ag(111) (calculated with PBE+vdW^surf^ for a single molecule in a 6 × 6 unit cell, as described in detail in Hörmann *et al.*)^282^ as a function of its distance (relative to the equilibrium distance). (b) Force per atom acting on the PTCDA molecule in *z*-direction.

In a geometry optimization, atomic displacements are performed in consecutive iterations until the forces acting on the atoms fall below a certain threshold. Generally, it is difficult to recommend good, generally valid thresholds (since this depends on the system). Nevertheless, it is instructive to consider a prototypical example, namely the adsorption of PTCDA on Ag(111). To simplify the discussion, we will not describe an actual geometry optimization, but discuss a simplified potential energy surface where the molecule was kept planar.^282^Fig. 19 shows the adsorption energy as a function of the adsorption distance together with its derivative in *z*-direction, *i.e.* the force pulling/pushing the PTCDA molecules towards/away from the surface. The displayed data were calculated with PBE+vdW^surf 161^ and were taken from Hörmann *et al.*^282^ (where also further information on the calculations can be found). Notably, the forces are below 10^−1^ eV Å^−1^ for essentially all displayed adsorption heights, *i.e.* stopping the geometry optimization when the (maximum) force per atom falls below 10^−1^ eV Å^−1^ would yield an uncertainty of about 0.10 Å on the adsorption height. Optimizations done with a threshold of 10^−2^ eV Å^−1^ (which are probably the majority of calculations in the literature at present) would here result in an intrinsic uncertainty of approx. 0.06 Å. This means that it would still not be appropriate to report adsorption distances with two or more significant figures behind the decimal point when using this convergence criterion. To obtain an accuracy of 0.01 Å, which is at or below the lowest experimental accuracies that can be achieved with modern structure characterization methods such as X-ray standing wave measurements,^328^ the remaining forces need to fall below 3 × 10^−3^ eV Å^−1^ (red region). Naturally, more accurate results can be obtained by choosing even tighter criteria, but this would also incur a dramatic increase in the number of geometry optimization steps required to reach this threshold.

It is important to emphasize that the connection between force threshold and accuracy of the geometry depends sensitively on the system and the method, *i.e.* the xc-functional and the employed van der Waals correction scheme,^176,282^ as they affect the corrugation and steepness of the energy landscape. The example here, PTCDA/Ag(111), is primarily bonded through dispersion, which shows a relatively shallow minimum. For systems which exhibit stronger molecule-surface interactions, a deeper and steeper minimum would be expected, *i.e.* smaller displacements could lead to larger forces.

To get a first idea about the intrinsic uncertainty of the adsorption height for a given force threshold, one can, in principle, start two different geometry optimizations with one starting guess notably above and the other notably below the expected adsorption height. However, when doing so one needs to be aware that between two geometry optimization steps, the molecule can move over substantial distances. As we discuss below for a different example, this is often >0.1 Å, *i.e.* similarly large as the basin itself. Therefore, at which point in the basin the optimization ends when employing this approach is, at least to some extent, arbitrary (*i.e.*, both optimizations could coincidentally end at very similar geometries or at the opposite boundaries of the basin). Thus, to get a reasonable idea about the associated error bar, one either needs to perform multiple geometry optimizations (and analyze the results statistically), or reduce the maximum distance each atom can move in one step. Alternatively, one can manually scan particularly sensitive geometrical parameters (like the adsorption distance in the above example) and then determine the distance at which the energy becomes a minimum from a fit (in analogy to performing a Birch–Murnaghan fit,^329^ that is used when calculating the optimum lattice constant in a bulk optimization).

Most practically relevant optimization schemes start by calculating the energy gradients with respect to displacements of the nuclei, *i.e.* the forces acting on each atom. The most straightforward method for geometry optimization would be to follow these gradients, *i.e.* move the atoms in the direction of these forces, until the gradients vanish. Such gradient-based methods, which indeed exist in multiple variants (steepest descent, conjugate gradient, *etc.*), are conceptually similar to linear mixing for the SCF inasmuch as they are guaranteed to converge, but are also computationally inefficient. Especially near the energetic minimum, gradient descent methods are prone to show oscillations, as the optimization will primarily follow “hard” degrees of freedom (where small displacements lead to large energy changes) rather than “soft” degrees of freedom (where large displacements have a modest impact on the energy). As a result, gradient-based methods produce good energies reasonably quickly, but may yield geometries where parts of the molecule are relatively far away from their correct positions.

Most modern codes instead rely on quasi-Newton optimization algorithms.^330–334^ In quasi-Newton methods a harmonic potential energy surface is assumed. This implies that the energy as function of the nuclear coordinates *E*(*Q*) can be described by a quadratic expansion around the energetic minimum (*E*_0_):7
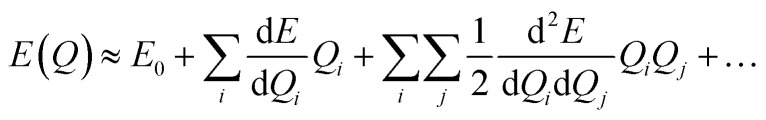
The first derivative of the energy with respect to the nuclear coordinates are the forces acting on the nuclei, while the second derivative is commonly termed Hesse matrix (or Hessian). In principle, if the assumption of a harmonic potential energy is valid and if the Hessian is known, the equation above can be inverted, and the minimum geometry is obtained within a single update. In practice, usually neither of the two conditions actually applies. In passing, we note that quasi-Newton schemes only update the inverse Hessian, as this is mathematically more efficient.

The assumption of a harmonic potential energy surface is only valid, when the structure is already close to the energetic minimum. This encompasses a relatively small fraction of the potential energy surface: Typically, the quadratic expansion is a reasonable model while atoms are within 0.05–0.20 Å of their equilibrium positions. Because the performance of quasi-Newton methods quickly deteriorates outside this region, for interfaces it is generally a good idea to put some effort into generating the initial (guess) structure, and to consider carefully, what the preferred adsorption sites and the most likely orientations of the molecule would be.

As a related issue, the harmonicity of a potential energy surface strongly depends on the coordinate system that is chosen. As so often for interfaces, the “optimal” choice differs for its chemically different constituents. For isotropically bonded materials with many bonding partners (metals and ionic materials), the movements of individual atoms are only weakly coupled. There, a simple Cartesian coordinate system (moving each atom in *x*,*y*,*z*) is sensible and effective. Conversely, for covalently bonded systems, such as molecules, the coupling is strong. Here, coordinate transformations to “internal coordinates” (which consist of bond lengths, angles, and torsions) are known to lead to much faster convergence.^247,335,336^ In practice, many band structure packages still perform their geometry optimization exclusively in Cartesian (or fractional) coordinates, but several “wrappers” exist that allow to circumvent this limitation.^337–340^ Although many programs are not very explicit about the choice of the coordinate system, it is strongly advised that the user finds out and takes the necessary caution when interpreting the results of a geometry optimization.

The more relevant challenge for quasi-Newton optimization schemes is that the Hessian (or its inverse) is not known *a priori*. While it could be calculated for the guess geometry, the computational effort for this is enormous: If calculated numerically, it requires 6 *N* evaluations of the forces on all atoms (where *N* is the number of unconstrained atoms, typically a few 10 to 100). This is as expensive as performing 6*N* geometry optimization steps. Even if “analytic” frequencies were used (employing density functional perturbation theory, which is available in a few code packages), this would still be computationally inefficient (despite a significant reduction of the necessary effort).

Therefore, the typically applied strategy is to start from an estimated Hessian as initial guess and to update it as the geometry optimization progresses. Following the common theme of interfaces, the optimal guess Hessians differs for different material classes. For the reasons discussed already in the context of choosing the coordinate system, isotropically bonded materials (metals/insulators) are commonly initialized using a scaled diagonal Hessian. This basically corresponds to assuming springs that bind each atom individually to its equilibrium position, neglecting any coupling between the atoms. Conversely, for covalently bonded systems, it is ideal to start from a Hessian in internal coordinates (bond lengths, angles, and dihedrals) and to choose the corresponding force constants based on pre-tabulated values (*i.e.*, based on a force field).^341,342^

**Fig. 20:**
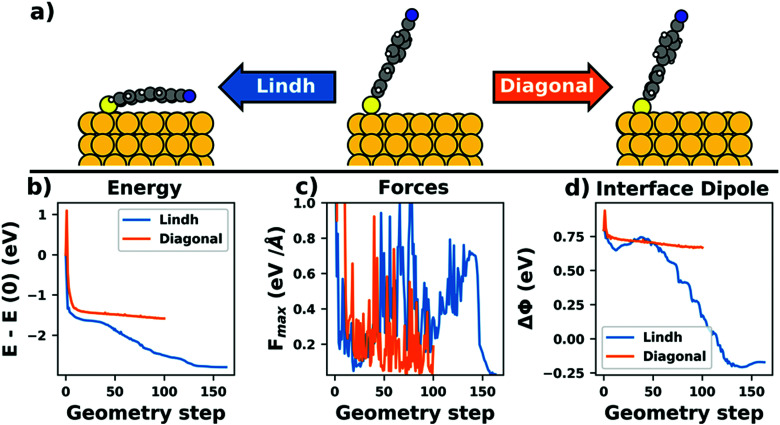
Geometries of a cyano-biphenylthiole molecule on Au(111), calculated in a 5 × 2√(3) unit cell with FHI-aims using the PBE+vdW^surf^ method and a 3 × 2 × 1 *Γ*-centered *k*-point grid. A three-layer metal slab was used. All atoms of the molecule and the topmost layer of the slab were allowed to relax until the remaining forces on all atoms fell below 0.01 eV Å^−1^. The calculations can be found on the NOMAD database. [https://dx.doi.org/10.17172/NOMAD/2020.12.07-9]. (a) Shows the starting geometry (center) and the final result after the optimization using a diagonal guess for the Hesse matrix (right) or using a Lindh guess (left). (b) Shows the evolution of the total energy relative to the energy of the starting point, for the two geometry optimizations. (c) Shows the remaining maximum force component *F* at each iteration step, (d) gives the interface dipole Δ*Φ*.

To illustrate the importance of the guess Hessian, we have calculated a low coverage monolayer of cyano-biphenylthiole on Au(111). As initial guess, we chose an almost upright, slightly tilted molecule, with plenty of space to each side, as shown in the central panel of Fig. 20. The optimization is performed in Cartesian coordinates. From a physics point of view we know that, triggered by van der Waals interactions, the molecule will “fall over”,^147^*i.e*., we expect a large change in the tilt angle during the geometry optimization. Thus, the initial structure is, geometrically speaking, quite far away from the actual minimum.

For our example we used two different initial guesses for the Hesse matrices, a scaled diagonal guess (which is the default in many codes) and a Lindh guess,^341^ which generates the guess based on pre-determined parameters for bond-lengths, angles, and dihedrals. In both cases, the geometry optimization was stopped when the remaining force on each atom fell below 0.01 eV Å^−1^, which is a typical, rather tight threshold employed in geometry optimizations. For the present example, the geometry optimization required more than 100 steps for both types of Hesse matrix initializations.

The resulting geometry for the diagonal Hessian is shown in the right panel of Fig. 20a. Only some small, short-ranged rearrangements of the atom positions have taken place (causing, *e.g.*, an increase of the inter-ring twist). However, the tilt angle of the molecular axis relative to the surface normal (identified before as the degree-of-freedom of primary importance) remains essentially unchanged. During the optimization, the total energy changes by slightly more than 1 eV relative to the energy of the initial geometry. Conversely, performing the same optimization using the Lindh-guess leads to much larger changes. Here, the energy decreased by almost 3 eV.

Geometrically, the most striking difference between the two geometry optimizations is the tilt angle relative to the surface. It is visibly different between the diagonal guess, where the molecule remains upright, and the Lindh guess (left part of Fig. 20a), where both rings are “in contact” with the Au surface. This is also reflected in the interface dipole Δ*Φ*, which, for the diagonal guess, remains at approx. 0.75 eV, but even goes to negative values for the optimization using the Lindh guess (see Fig. 20d).

Since the two geometry optimizations exited gracefully, the two resulting geometries could easily be interpreted as being two separate minima. However, it is unlikely that this is indeed the case. Had we continued the optimization to an even tighter threshold, even with the diagonal guess the optimization would eventually find the same minimum structure – however, very inefficiently, making the associated computational effort too large to be tractable. We can backtrack the Lindh optimization and ask what would have happened, had we used a lighter threshold: Had we chosen to run the optimization until the remaining forces fell below 5 × 10^−2^ eV Å^−1^ (which is, *per se*, not unreasonable for large systems), also the optimization with the Lindh Hesse matrix would have finished with a de-facto upright standing molecule.

The present and the previous example illustrate two important messages: (1) Even when a calculation converges nicely, it is important to critically second-guess the resulting geometry. (2) It is important to use sufficiently tight thresholds. The “ideal” value of the threshold depends on the physics of the system, the required accuracy of the geometry, and also the employed geometry optimization scheme and the employed parameters (such as the values for the guess Hessian in the present example).

**Fig. 21:**
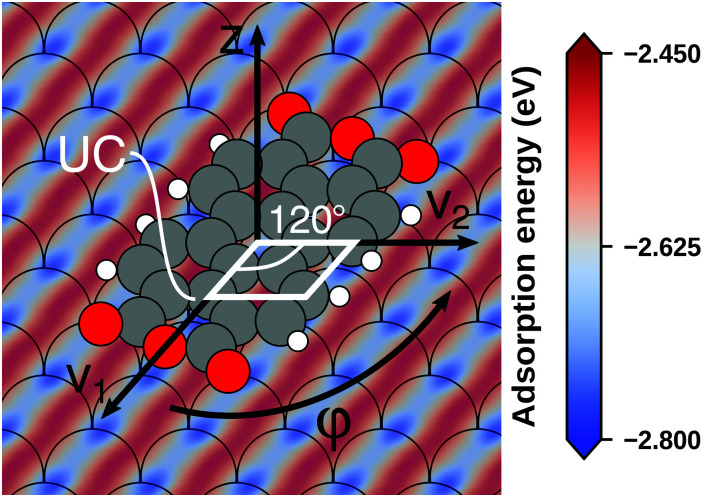
Potential energy surface for the translation of a rigid PTCDA molecule 2.9 Å above the Ag(111) surface, calculated for a 6 × 6 supercell using PBE+vdW^surf^, as obtained by Hörmann *et al.*^282^ v1 and v2 denote the primitive lattice vectors of the substrate, *z* the adsorption height and *φ* the rotation angle of the molecule relative to the substrate. The potential energy surface shows a minimum on the Ag-bridge site, a low-energy saddle point along v1 and a high-energy saddle point along v2.

It is also instructive to briefly consider why the optimization proceeds so slowly here (convergence only after more than 100 steps), even when using the Lindh-matrix as initialization. Three factors play a major role: First, as mentioned above, the interactions that primarily cause the molecule to tilt are van der Waals interactions. These are, however, not contained in the Lindh parameterization. Secondly, because van der Waals forces decay very quickly with distance, they “pull” more efficiently on the bottom ring than on the top ring. This reduces the overall tendency of the molecule to change its tilt. The major factor is, however, the maximum step size. All geometry optimizations employ a maximum distance by which a given atom can move between subsequent steps (typically about a tenth of an Å; for Fig. 21, the default value of 0.2 Å was chosen), in order to avoid creating unphysical structures with broken bonds. Here, for the molecule to fall over, the nitrogen atom has to move by several Å. Even in the most ideal case, the step size limit (for which, again, the default in the present example was clearly suboptimal) requires several dozens of steps to allow reaching the equilibrium structure. This problem can be mitigated by increasing the corresponding parameter in the input, or by using dedicated optimization tools (*e.g.*, GADGET^337^).

The efficiency of quasi-Newton schemes for interfaces is, however, fundamentally limited by the physics of the interface. In quasi-Newton optimization schemes, the effective step size is always determined by the smallest eigenvalue of the inverse Hessian (the stiffest mode, since this leads to the largest energy change), while the maximum move occurs along the softest mode (described by the lowest eigenvalue of the Hessian). In other words, the convergence speed depends on the ratio of highest to lowest eigenvalue of the Hessian (the so-called “condition number”):^337^ the higher the ratio, the slower the convergence. Although coordinate transformations can favorably shift the condition number^337^ (*e.g.*, by going from Cartesian to internal coordinates), at interfaces, it will remain intrinsically high. The large substrate cell contains some very soft modes originating from a backfolding of the acoustic modes of the primitive substrate unit cell. Also intermolecular interactions are very soft, and so is the (often van der Waals driven) interaction between substrate and adsorbate. Therefore, the maximum move occurs along these modes. Conversely, covalent bonds are very stiff (particularly double and triple bonds with the CN-bond in the above example as the prototypical “worst case” scenario), determining the step size. Because this broad distribution of modes is a fundamental property of interfaces, which also does not change when using a different coordinate system, even optimizations that work in internal coordinates would be slow here.

It is important to stress that the described behavior is quite general for interfaces, and not limited to the somewhat extreme biphenylthiole example from above, which has been chosen also because it illustrates the problems particularly clearly. Other examples where convergence is slow include cases in which the molecule ought to rotate on the surface to reach a minimum structure. There, the necessary significant changes in the Hessian matrix are not easily captured by the update algorithm when working in Cartesian coordinates, resulting in very slow convergence. A similar problem occurs when forces on individual atoms are small, but all point in the same direction, *i.e.* when the molecule translates.

A further peculiarity of quasi-Newton optimizers to keep in mind is that, unlike most other geometry optimization schemes, they are not guaranteed to follow the direction of the gradient downward in energy. Rather, the optimization will proceed to the closest stationary point, which can also be a saddle point, if the present guess geometry is sufficiently close to it (*i.e.*, in its “harmonic” region).^292^ For interfaces, this is mostly an issue for degrees of freedom that move unbounded between symmetry equivalent positions, such as translation across the surface or rotation of the adsorbate, as shown in Fig. 21. For these motions equivalent minima are separated by a saddle point. For reasonably complicated adsorbates, there is little chemical intuition where the minimum should be, making it likely for novice (and even veteran) computational scientists to start the geometry optimization near a saddle point.

To reduce the chances of landing on a saddle point, it is possible to pre-optimize the geometry with a different method, such as steepest descent. This is guaranteed to follow the direction of the gradient. It is furthermore a robust method for pre-optimizations when the geometry is far away from the harmonic region of the potential energy surface. Therefore, Conjugate Gradient preoptimizations are especially advisable when there is little intuition about the potential energy surface and the positions of the energetic minima.

We note that also other geometry optimization algorithms exist that sometimes may be superior to quasi-Newton methods. Since these are not very widespread (yet), here we would like to mention only two: damped molecular dynamics (DMD) and Bayesian Optimization.^166,343^ Both have the advantage of not relying on a harmonic expansion, *i.e.* they should perform well also far away from the minimum. In our experience, DMD at interfaces suffers from the simultaneous presence of soft and hard modes: Hard modes necessitate small timesteps, since otherwise the molecule breaks up (which happens frequently, *e.g.*, when CN groups are involved), but too small timesteps make it harder for the molecule to move efficiently along soft modes. Overall, while DMD with optimized parameters may show a faster convergence than quasi-Newton methods ever will, finding these parameters can be tricky and (unless good values are known beforehand) is, in our experience, hardly worth the effort.

Bayesian Optimization is a relatively new method, for which not much experience has been accumulated so far in the context of surface science. Generally, Bayesian Optimization techniques work well when there are only few degrees of freedom, but the computational effort becomes too large for larger systems.^344^ Explained in a very simplified way, Bayesian Optimization relies on calculating a few selected geometries and constructing a (conditional) probability distribution for the energy of other geometries, *i.e.* effectively interpolating between them. In contrast to the other optimization methods, it can, therefore, provide a general (at least qualitative) overview of the potential energy surface, including the positions and locations of the minima and saddle points. We presently recommend to employ Bayesian Optimization following the strategy described by the Rinke group:^349^ Keep the internal structure of the adsorbate (bond lengths, angles, *etc.*) and the substrate fixed, only moving and rotating the molecule as a whole across the surface. This procedure allows to quickly identify the attraction basins of different local minima on the surface. Moreover, it provides a good overview of (but certainly no exact result for) the correct values for these soft degrees of freedom. The obtained geometries are then used as starting points for quasi-Newton optimizations, which then find the minimum structures (usually) very efficiently. In this case, the Bayesian Optimization replaces the above-mentioned preoptimization with conjugate gradient and additionally allows a more systematic screening of the entire potential energy surface, revealing several local minimum structures.

**Fig. 22:**
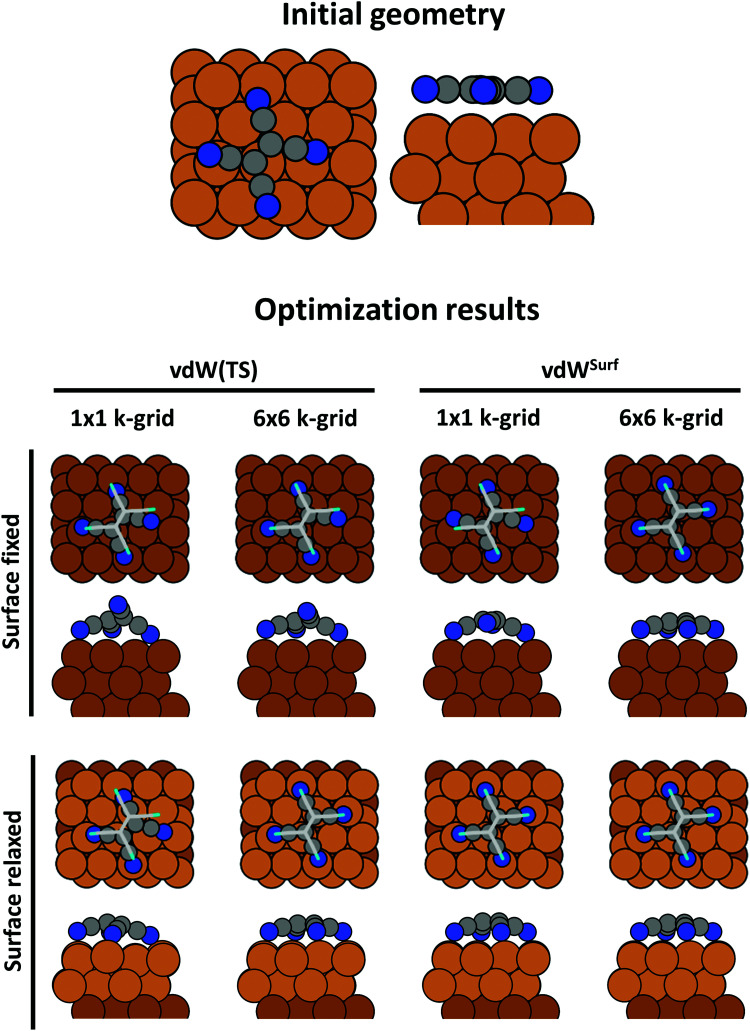
Geometry optimizations of a flat lying TCNE molecule on Cu(111) with varying computational strategies: changing *k*-point densities, different van der Waals corrections and fixing the full substrate *vs.* relaxing the two uppermost layers. The grey wireframes indicate the adsorption position obtained with the most elaborate strategy (relaxed surface, 6 × 6 *k*-points and vdW^surf^ correction). Dark Cu atoms were fixed, lighter Cu atoms were allowed to relax during the geometry optimizations. All calculations were done with FHI-aims. Additional details can be found in https://dx.doi.org/10.17172/NOMAD/2020.12.10-1.

Besides the different geometry optimization strategies mentioned here, also other settings in the calculation can influence the optimized geometry. We illustrate this for the example of TCNE on Cu(111). There, we performed various geometry optimizations starting with a flat lying molecule from the same, randomly (*i.e.*, deliberately, not intentionally) chosen position while varying (i) van der Waals corrections, (ii) *k*-points, (iii) substrate relaxation strategies (fixing the whole substrate *vs.* optimizing the two uppermost Cu layers). Fig. 22 illustrates the geometry optimization results for all combinations of settings. When fixing the substrate and using a vdW correction without surface reparameterization, the TCNE molecule only binds with two C=N groups while the others are bent away from the surface. Introducing a surface-parametrized vdW correction partly prevents this behavior due to the now stronger adsorbate-surface interaction, but there are still differences between the optimization with a single *k*-point only and a well converged *k*-grid (different adsorption site and bending). Optimizing the uppermost substrate layers makes the situation more reproducible, at least in the considered case. Here the surface parametrized vdW correction leads to identical results independent of *k*-point density and also without surface parametrization the “correct” adsorption geometry is found with the 6 × 6 *k*-point grid. This shows that for reliable results, not only the geometry optimization algorithm must be chosen suitably, but also the other methodological choices (discussed earlier in this manuscript) need to be chosen appropriately.

**Fig. 23:**
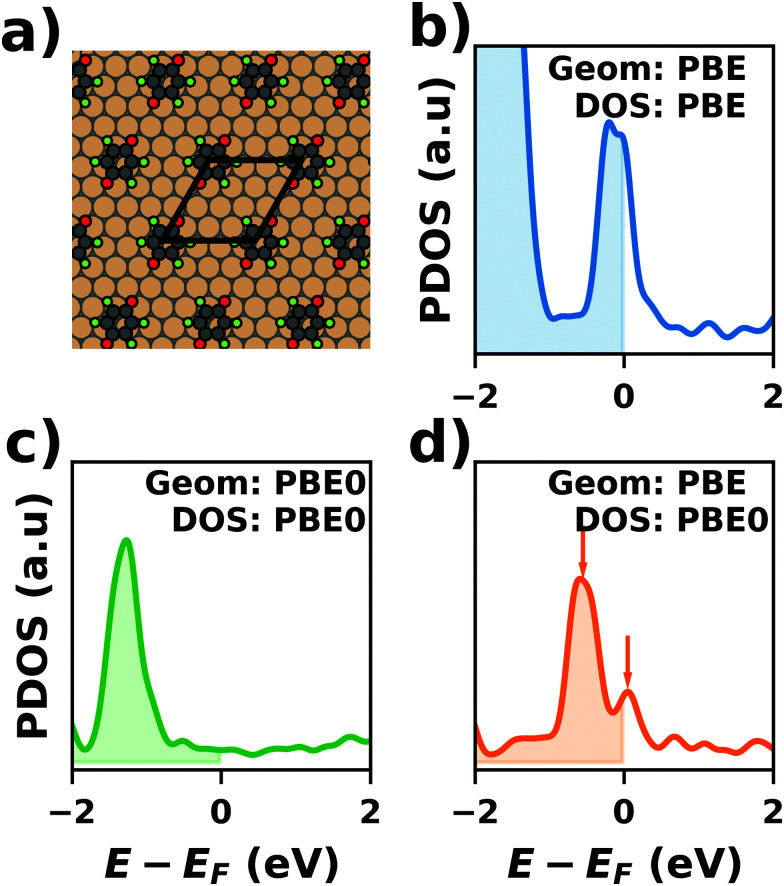
Projected densities of states (PDOS) of tetrafluorobenzoquinone in a *p*(4 × 4)Cu(111) cell projected onto the adsorbate layer. (a) Is a representation of the geometry in a 2 × 2 supercell. (b) Shows the result for the projected DOS obtained with PBE after also optimizing the geometry with PBE. For the results in (d) the same geometry was used, but the electronic structure of the interface was recalculated using PBE0. For (c) PBE0 was used for re-optimizing the geometry and for calculating the electronic structure of the interface. All calculations were done using FHI-aims and a 6 × 6 *Γ*-centered *k*-point grid. All geometry optimizations were performed including the vdW^surf^ correction. For further details see Wruss *et al.*^348^ and the calculations in the NOMOAD database at 10.17172/NOMAD/2020.12.11-2.

Before we conclude the section on geometry optimization, we want to briefly comment on the possibility of optimizing the geometry at a given level of theory, while then performing a single-point calculation on that geometry at a higher level of theory to obtain a supposedly more accurate electronic structure of the interface. Due to the high cost of geometry optimizations, this is a tempting scheme, which is frequently applied in quantum chemistry. For interfaces, this usually means that the geometry is obtained using a dispersion-corrected semilocal functional (*e.g.*, PBE), and then the electronic structure is computed with a more expensive hybrid functional (*e.g.*, PBE0). In most cases, this approach works well and is very useful when trying to gain a more quantitative insight. However, because electronic and geometric structure are strongly coupled, this approach bears the risk of running into serious artefacts, especially when the two methods predict qualitatively different electronic structures of the interface. Obvious manifestations of qualitatively different solutions occur, when the application of hybrid functionals leads to a strong localization of the electrons,^13^ when the order of occupied and unoccupied states swap,^43,103^ or when different spin-configurations are obtained by the two methods.^308^ A less obvious, but nonetheless salient difference can occur when modelling the adsorption of flat-lying conjugated organic molecules undergoing significant charge transfer with the substrate. This is demonstrated in Fig. 23 for the adsorption of tetrafluorobenzoquinone (TFBQ) on Cu(111).^237^

Here, calculating the projected density of states for the adsorbed molecule using PBE+vdW^surf^ for both the geometry and the electronic structure (shown in Fig. 23b) shows a partially filled molecular feature at the Fermi energy, *i.e.*, a metallic character of the adsorbate.^345^ Re-calculating the electronic structure at a higher rung, *i.e.* with the hybrid functional PBE0 (Fig. 23d) yields an electronic structure that is similar inasmuch as it still contains a partially filled adsorbate-derived feature at the Fermi-energy. The situation is still qualitatively different, since now the projected density of states exhibits a clear double peak structure (indicated by arrows). Following the Newns–Anderson model^346^ (and, in fact, much of established chemistry), such double-peak structures are characteristic for the formation of bonding and antibonding states due to strong chemisorption, *i.e.* the formation of a new, partially covalent bond.

However, when also optimizing the geometry at the PBE0+vdW^surf^ level, the density of states near the Fermi-energy vanishes, as does the double peak structure (see Fig. 23c). In other words, the interface chemistry is completely different: The adsorbate is no longer metallic, *i.e.* no longer exhibits a fractionally filled state, the LUMO-peak moves to lower energies and loses its double-peak structure, indicating that the bond between substrate and adsorbate is predominantly of ionic, not covalent, character. This behavior is not a peculiarity of the TFBQ molecule. Rather, it originates from the fact that semilocal and hybrid functionals yield notably different bond lengths for single and double bonds.^347^ Consequently, when performing a hybrid functional calculation with a semilocal geometry, the individual bonds are out of their equilibrium, which makes the system too reactive, triggering a tendency to form new, covalent bonds.

## 6 Summary

In this work, we discuss various relevant technical, mathematical, physical, and chemical aspects that need to be considered when performing state-of-the-art first-principles simulations on hybrid inorganic–organic interfaces. Those interfaces pose a significant challenge, since they display strongly varied physics: they contain covalent, metallic, ionic, hydrogen and van der Waals bonds, often all at the same time. Charge transfer, localization, and the emergence of collective electrostatic effects that shift the relative level alignment of substrate and adsorbate complicate the electronic structure. Due to the different bond types, interfaces contain very hard and very soft degrees of freedom. All of these effects need to be accounted for accurately. This is often a considerable challenge due to the large size of the systems used to model such interfaces, which makes the simulations computationally very demanding. Therefore, we discuss different methods to model hybrid inorganic–organic interfaces, study the choices and reasoning behind different ways to construct atomistic models of the interfaces, and debate the impact of a variety of choices concerning the numerical algorithms and parameters employed in calculating the electronic structure and in performing geometry optimizations. Throughout this review, we highlight the advantages and disadvantages of different approaches in order to provide the reader with the basis to make educated and informed choices. We provide some general “best practice rules” throughout the work, which are summarized in Table 1. We emphasize that these rules reflect our current knowledge – they are certainly not complete, nor are they necessarily completely universal.

**Table 1:** Suggested “best practice” rules for interface simulations

Best practice rules for interface simulations
**The structural model**
• Open *versus* periodic boundary conditions: cluster calculations with proper embedding and slab calculations (with appropriate supercells) give conceptually equivalent results when properly converged. Clusters can be more efficient when modelling inherently non-periodic or diluted effects (*e.g.*, defects or excitations). Slab calculations are typically easier to converge.
• When using periodic boundary conditions, employ a dipole correction or a Poisson solver for mixed boundary conditions.
• Make sure the position of the vacuum level is further away from the ends of the slab than the separation between the dipoles (*i.e.*, more than the length of the lattice vectors in *x* and *y*)
• When calculating charged unit cells, use a charge compensation scheme (CREST, metallic boundary conditions, generalized dipole correction, *etc.*)
• Periodic boundary conditions often require stretching or compressing the unit cells of adsorbate or substrate to obtain a common (commensurate) unit cell. Test carefully that this scaling does not affect the electronic properties.
• There are often many local minima for the adsorption of an organic molecule on a surface. Explore the potential energy surface sensibly, if necessary, by using multiple starting points for geometry optimizations, preferably by a global structure search algorithm, such as Gaussian Process Regression or Genetic Algorithms.
• When simulating an interface, use at less than 4 layers for the substrate only in well-justified circumstances. If feasible, converge the number of layers for the property of interest.
• When optimizing adsorbate geometries, relax at least the two topmost substrate layers; keep half of the slab or more constrained to speed up the geometry optimization and reproduce bulk-like behavior for the slab.

**The electronic structure method**
• Van der Waals forces are very important for interface systems. Always use a van der Waals-inclusive method (either couple with a correction scheme or use a non-local functional)!
• Different functionals will yield different results. Resist the urge to vary functionals just to obtain the desired results. Try to understand why one functional provides the desired result and another one doesn’t.
• Understand numerical limitations of the electronic structure method. Metal–organic interfaces are typically well described with semilocal functionals. Semiconductor–organic interfaces require (tuned) hybrid functionals and a method to account for doping.
• Obtain geometry and electronic structure at the same level of theory if possible. If not, be aware that even small changes of the geometry may affect the interface physics/chemistry.
• For systems with large interface dipoles, use a linear mixer with a small mixing coefficient before switching to the Pulay mixer.
• Use relatively small coefficients for preconditioner and mixing. Very small preconditioner values reduce the impact of the mixing parameter.
• Start with a reasonably large broadening parameter (∼0.1–0.2 eV), verify results with a smaller value (∼0.01) and a denser *k*-point mesh.

**Numerical settings and algorithms**
• Be aware which numerical accuracy for which property is desired. Choose numerical settings tight enough to converge a property to a meaningful numerical accuracy. Any tighter and you waste computational time. Any looser and you generate underconverged results.
• Always converge the settings of the calculations for the property of interest. This is not always the total energy! Monitor convergence for each desired property explicitly.
• Check for spurious convergence by inspecting the evolution of the SCF. Suddenly reaching the requested threshold is suspicious.
• Converge, converge, converge! Converge your numerical settings, but also converge the thresholds for the SCF. Convergence tests are time-consuming and boring, but they are the only way to reliably obtain accurate and robust results.
Understand the defaults of the software package you are using.
• The convergence behavior differs between similar systems – do not lightheadedly adopt settings developed for another system.

**Geometry**
• Get an overview of the potential energy surface, *e.g.* by using Gaussian Process or Bayes Linear regression. Start geometry optimizations near expected minima.
• Create good guess structures before starting the geometry optimization; ideally, multiple guess structures should be used to obtain an idea about the reproducibility.
• Optimize in internal coordinates if possible; be aware of the initialization of the guess for the Hesse matrix; avoid diagonal matrices if possible.
• For systems that are (partially) open-shell, break the spin-symmetry by assigning non-zero initial spin to the atoms.
• When optimizing geometries, be aware of the limits of the force thresholds. If possible, map out important degrees of freedom explicitly to determine the minimum accurately. Alternatively, start multiple geometry optimizations from different starting points.

It is just as important to be aware of the many possible algorithms and options that can be chosen to calculate the electronic structure. We lay the focus of this work on illustrating some of the pitfalls that exist when using the default values and algorithms that pertinent codes use. Based on several examples – some from our previous work, but most specifically calculated for this work – we show how the application of inappropriate defaults or other sub-optimal settings can easily lead to quantitatively or also qualitatively incorrect results. Our account is based on personal experience and the current state-of-the-art of available methods. We expect (and indeed hope) that some of the best practice suggestions might become obsolete in the future as more advanced methods become available. We hope that the lessons contained in this work, at the very least, will help to spot spurious results in literature and to avoid falling for artefacts in one's own work.

At the same time, we try to suggest sensible values and default values for interface simulations, that will not only provide correct results, but will also allow to develop faster and more efficient computational workflows. Of course, every interface is special, and those defaults will not always be optimal. We do our best to explain the reasoning for those values and emphasize the correlation with the underlying physics of the interface, to allow the reader to tailor these suggestions to their own system.

## Conflicts of interest

There are no conflicts to declare.
